# Spontaneous Emulsification as a Low-Energy Strategy for Designing and Optimizing Resveratrol-Loaded Nanostructured Lipid Carriers

**DOI:** 10.1021/acsomega.5c10613

**Published:** 2026-02-11

**Authors:** Nicoly T. R. Britto, Lilian R. S. Montanheri, Juliane N. B. D. Pelin, Tereza S. Martins, Patrícia S. Lopes, Vânia R. Leite-Silva, Newton Andreo-Filho

**Affiliations:** † Department of Pharmaceutical Sciences, Federal University of Sao Paulo, Diadema 09913-030, São Paulo, Brazil; ‡ Department of Chemistry, Federal University of Sao Paulo, Diadema 09913-030, São Paulo, Brazil; § Therapeutics Research Centre, The University of Queensland Diamantina Institute, Translational Research Institute, Brisbane, Queensland 4102, Australia

## Abstract

Nanostructured lipid carriers (NLCs) have proven to be effective for the delivery of lipophilic drugs by combining high encapsulation capacity with controlled release properties. In this study, NLCs were developed using spontaneous emulsification, a low-energy, simple method that is free of organic solvents. Cetearyl alcohol and Kolliphor RH 40 were employed as the solid lipid and surfactant, respectively. The formulation and process were optimized through a Box–Behnken design, considering mean particle size and size uniformity, as determined by laser diffraction. Colloidal stability was improved by the addition of sodium lauryl sulfate and adjustments to the dispersion polymer. Resveratrol (RVL), used as a model bioactive compound, achieved an encapsulation efficiency of 99%, with a mean particle size of 90 nm, a polydispersity index of 0.2, and a zeta potential of −37 mV, remaining stable for 90 days at 8 °C. Characterization by differential scanning calorimetry/thermogravimetry, Fourier transform infrared, and transmission electron microscopy confirmed the incorporation of the active compound as well as the composition and morphology of the nanocarriers. The system exhibited a delayed and sustained release of RVL, highlighting its potential as a platform for the delivery of bioactive compounds. Overall, the results demonstrate that spontaneous emulsification is a viable and sustainable alternative to the high-energy methods predominantly reported in the literature, enabling the production of stable and efficient NLCs for pharmaceutical and cosmetic applications.

## Graphical abstract

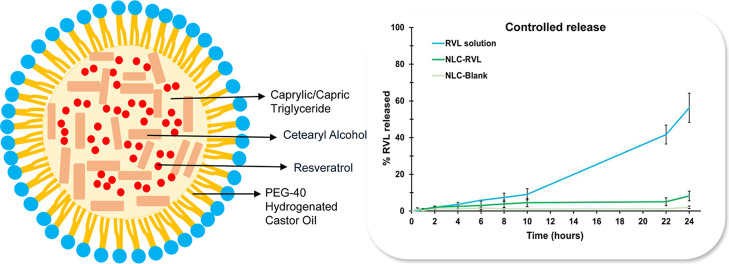

## 1 Introduction

Nanotechnology has driven remarkable advances across various scientific fields due to the unique properties of materials on the nanoscale. This progress has sparked growing interest from both the scientific community and the industrial sector, each seeking to enhance well-being and quality of life.^1^ In the biomedical field, for example, nanostructured systems have been extensively investigated for applications ranging from controlled drug delivery to the precise diagnosis of pathologies.^2^

Among the wide range of nanostructured platforms explored, lipid-based nanostructured systems—including microemulsions, solid lipid nanoparticles (SLN), nanostructured lipid carriers (NLCs), and nanoemulsions—stand out as promising vehicles for the delivery of bioactive compounds in cosmetic, food, and therapeutic applications.^2,3^ The formation of these systems relies on a strategic combination of formulation composition and manufacturing process to enable the dispersion of oil droplets, with diameters between 50 and 1000 nm, in a continuous aqueous phase.^4^

In this context, nanoemulsions are predominantly composed of liquid lipids, resulting in dispersed liquid droplets in an aqueous medium at room temperature.^5^ In contrast, SLNs consist exclusively of solid lipids with melting points above room or body temperature, which confer a solid state to the particles.^6^ NLCs, on the other hand, represent a hybrid system positioned between nanoemulsions and SLNs. Although their particles have melting points above body temperature, their lipid matrix is a mixture of solid and liquid lipids in variable ratios.^7^ The diversified lipid matrix composition directly influences critical properties, such as drug-loading capacity, release kinetics, interaction with the biological environment, and the physicochemical stability of the system.^8^

Beyond composition, the manufacturing process plays a decisive role in obtaining successful lipid-based nanostructured systems. Broadly, production methods can be classified into two main approaches: top-down and bottom-up.^9^ In the top-down approach, larger structures, such as conventional emulsions, are subjected to high-energy processes, including high-pressure homogenization, microfluidization, and ultrasonication. These methods apply intense cavitation, high pressure, and high temperature to break down larger droplets into nanoscale droplets. Although widely employed in both research and industry for producing lipid nanocarriers, these processes require specialized equipment, which can result in high production costs.^9–11^ In contrast, bottom-up processes adopt the opposite strategy, generally starting from molecular dispersions of lipids in organic solvents and promoting the self-assembly of these lipids into droplets or particles, typically through solvent removal by evaporation or transfer into the aqueous phase.^12,13^

In both cases, the production of nanostructured systems may involve numerous process variations. However, these often require specialized equipment or large volumes of organic solvents, necessitating formulation purification steps. Such requirements can extend the production time and increase associated costs. In this context, the development of simplified processes for obtaining lipid-based nanostructured carriers is of great interest to both industry and society. Overcoming the challenges associated with scaling up nanostructured systems from laboratory research to industrial production—without relying on specialized equipment such as high-pressure homogenizers, sonicators, and microfluidizers, which differ substantially from the common processing equipment found in production lines, such as homogenization and heating tanks—as well as avoiding the need for technical infrastructure or organic solvents that require industrial facilities for containment, recovery, reuse, and environmental monitoring of formulation residues—represents a critical research challenge with a direct impact on the feasibility and accessibility of innovative therapies and products.

Accordingly, this study proposes the development of NLC via spontaneous emulsification, a low-energy method that eliminates the need for specialized equipment and organic solvents. While this method is commonly employed for the preparation of nanoemulsions, its application for NLC development remains largely unexplored. Furthermore, both the process and formulation were optimized by using a Box–Behnken design (BBD) experimental plan, with significant factors identified through response surface methodology (RSM) analysis. To assess the intrinsic capacity of the method and formulation to encapsulate bioactive compounds, resveratrol (RVL) was selected and incorporated as a model bioactive, providing a robust basis for validating the applicability of the proposed approach.

The development strategy adopted in this study is illustrated in the flowchart presented in Figure 1.

**Figure 1:**
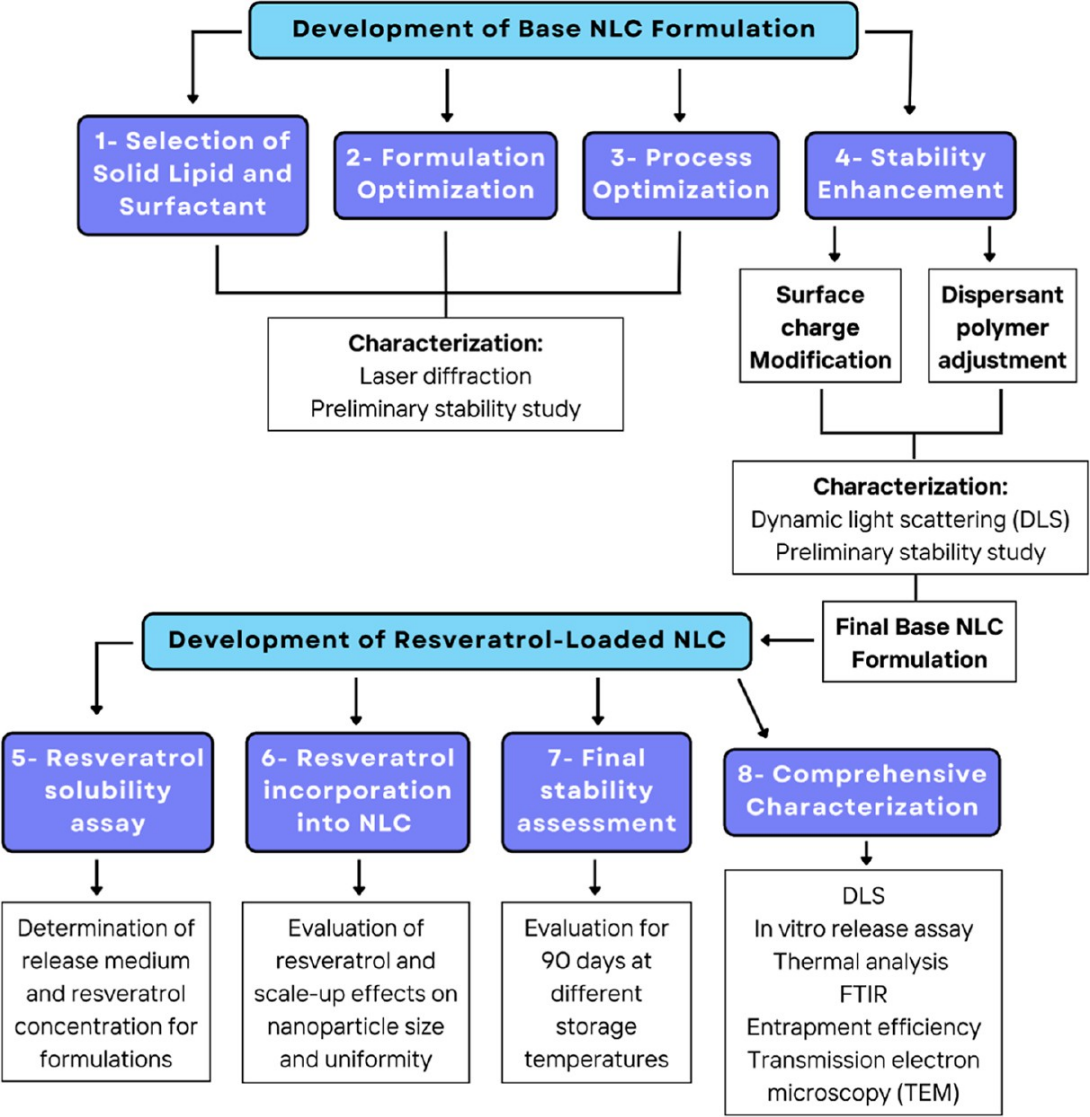
Flowchart of the development and evaluation steps of the NLC formulation.

## 2 Materials and Methods

### 2.1 Materials

Oleth-20 (O20), Ceteareth-20 (C20), Caprylic/Capric Triglyceride (CCT), cetearyl alcohol (CA), glyceryl stearate (GS), cetrimonium chloride (CTAC), and sodium lauryl sulfate (SLS) were purchased from Aqia (Guarulhos, Brazil). PEG-40 hydrogenated castor oil (CORH40), polyvinylpyrrolidone K30 (PVP), and poloxamer 188 (P188) were purchased from BASF (São Paulo, Brazil) and RVL from DSM (Campinas, Brazil). Disodium phosphate dodecahydrate (Na_2_HPO_4_12H_2_O), sodium phosphate (NaH_2_PO_4_), and polysorbate 80 (Tween 80) were purchased from Labsynth (Diadema, Brazil), and ethanol (≥99.5%) was purchased from Dinâmica Química (Indaiatuba, Brazil). Purified water used in all experiments was obtained by reverse osmosis (OS10LXE, Gehaka).

The components used in the formulation are classified as GRAS, approved, and commonly employed in topical (cosmetic) preparations. In addition, they are used in relatively small amounts, which means that the final formulation would not pose safety concerns. These excipients are also biodegradable and are not expected to accumulate in the body or in the environment. We acknowledge that CTAC and SLS may be irritating when used topically; however, in our study, they were incorporated only at minimal concentrations, solely to adjust surface charge.

### 2.2 Preparation of NLC Formulation

The formulations were prepared by using the spontaneous emulsification method. Initially, the components were weighed separately into two phases (aqueous and oily). The oily phase was melted in a water bath at 65 °C, while the aqueous phase was heated separately to 80–90 °C. Once the target temperatures were reached, the aqueous phase was added dropwise to the oily phase under magnetic stirring, resulting in a translucent dispersion. The formulation was maintained under stirring at room temperature until the cooling was complete.

### 2.3 Selection of Solid Lipid and Surfactant

In this stage, six formulations were prepared, varying the solid lipid (2%): GS or CA, and the surfactant (5%): Oleth-20 (O20), Ceteareth-20 (C20), or PEG-40 hydrogenated castor oil (CORH40). The general composition of the formulations included the liquid lipid CCT (2%) and an aqueous phase consisting of 50% of PVP solution (2%) and water q.s to 100%. The formulations were analyzed using laser diffraction. The parameters obtained from this analysis guided the selection of raw materials, establishing the following criteria: percentage of particles with a diameter equal to or smaller than 500 nm (% *p* ≤ 500 nm) greater than 75%, mean diameter below 500 nm, SPAM (dispersion index) below 3, and uniformity ratio (UR) below 10. Formulations that met these parameters were prepared again to assess the reproducibility. They were subsequently subjected to a preliminary stability study for 2 days at room temperature, during which the same analysis was performed.

### 2.4 Formulation Optimization

To optimize the formulation composition, a design of experiments (DoE) based on the Box–Behnken design (BBD) approach was conducted using Minitab software (version 18.1). The independent variables considered for formulation optimization were surfactant concentration ([surfactant]), proportion of PEG-40 hydrogenated castor oil (CORH40) [with the difference compensated by Ceteareth-20 (C20)], and total oily phase concentration ([oily phase]), each evaluated at two levels (+1 and −1) with a central point (0). The dependent variables were particle size distribution (PSD) parameters obtained by laser diffraction: % *p* ≤ 500 nm, mean particle diameter, SPAM, and UR.

The formulations considered within the acceptance criteria were those that met the following parameters: more than 90% of particles ≤500 nm, mean diameter between 150 and 350 nm, SPAM below 3, and UR below 5. Data were analyzed using RSM to identify the effects of the independent variables related to formulation composition on the particle size and size uniformity parameters of interest. For formulation optimization, the target values were set as follows: 99% *p* ≤ 500 nm, mean diameter = 190 nm, SPAM = 1.65, and UR = 3.9.

The optimized formulation was analyzed by laser diffraction at 0, 1, 2, 7, and 15 days to monitor particle size and size uniformity stability over time.

### 2.5 Process Optimization

A second experimental design was conducted to optimize the processing conditions. The independent variables were: oily phase temperature (°C), aqueous phase temperature (°C), stirring speed (rpm), and the order of phase addition (oily into aqueous or aqueous into oily). The dependent variables (responses) were the same as those considered for formulation optimization: % *p* ≤ 500 nm, mean particle diameter, SPAM, and UR, along with the corresponding target parameters. Data were analyzed using RSM, and process optimization was performed based on the predefined targets: 95% *p* ≤ 500 nm, mean diameter = 200 nm, SPAM = 2, and UR = 3.

The final optimized formulation was reproduced in a scaled-up batch (10 times the amount of the initially prepared formulation) to assess reproducibility and the stability of particle size and size uniformity, as well as the process efficiency regarding formulation scale-up. The formulations were monitored over 15 days to evaluate their stability.

### 2.6 Stability Improvement

#### 2.6.1 Surface Ionic Charge Addition

Two ionic surfactants—one anionic, SLS, and one cationic, CTAC—were added at concentrations of 0.05, 0.1, and 0.2% to the aqueous phase of the optimized formulation to assess the influence of surface charge on parameters related to particle size, extension of the stability period, and improvement of the formulations appearance.

The formulations were stored at room temperature, protected from light, and analyzed by dynamic light scattering (DLS) for particle size, polydispersity index (PDI), and zeta potential (ZP) at 0, 7, and 14 days after preparation to evaluate stability.

#### 2.6.2 Modification of the Dispersing Polymer

The dispersion polymer or its concentration was adjusted to increase viscosity, thereby reducing nanoparticle mobility and aggregation, which consequently extended the particle size stability period and maintained the initially obtained PDI values.

For this purpose, three formulations were produced by modifying the aqueous phase:

- F1: 50% aqueous phase PVP 2%, q.s. water to 100%.
- F2: 50% aqueous phase PVP 10%, q.s. water to 100%.
- F3: 50% aqueous phase PVP 2%, q.s. P188 2.5% to 100%.

It is important to note that these were the only modifications made with no changes to the remaining composition (raw materials or concentrations) or to the process, which remained as defined up to the addition of the ionic surfactants.

The formulations were analyzed by DLS at 0, 7, and 14 days after preparation to monitor the stability. Samples were stored at room temperature and protected from light.

### 2.7 RVL Solubility Assay

The solubility of RVL was tested in six different solutions to select one for subsequent use in the release assay. The solutions were composed of phosphate-buffered saline (PBS) and absolute ethanol (EtOH) or Tween 80 (Tw).

For each test, 6 mg of RVL was weighed into separate vials, and 20 mL of each solution was added (0.3 mg/mL), corresponding to 10 times the solubility of RVL in water (0.03 mg/mL).^14^ The 50 mM PBS solution (pH 7.4) was prepared by using 2.6 g of monosodium phosphate (NaH_2_PO_4_) and 26.8 g of disodium phosphate dodecahydrate (Na_2_HPO_4_·12H_2_O).

The vials were incubated at 32 °C under 200 rpm agitation for 48 h. After incubation, samples were centrifuged at 5000 rpm for 15 min at 25 °C. The supernatant was filtered through a syringe filter (hydrophilic PTFE membrane, 0.45 μm pore size) and subsequently diluted for quantification by spectrophotometry at 306 nm. The solution exhibiting the highest RVL solubilization concentration was selected as the receptor medium for the release assay.

Additionally, to determine the RVL concentration to be incorporated into the formulations, solubility tests were performed in the oily phase, composed of CORH40, CCT, and CA in amounts corresponding to 20 g of the formulation. The oily phase was first heated in a water bath at 65 °C to melt the lipids and then placed on a magnetic stirrer at 70 °C with 700 rpm agitation. Next, 100 μL of a 5% RVL solution in ethanol was added and stirred for 5 min, followed by 5 min at rest in the water bath. This procedure was repeated twice, adding 20 μL of the RVL solution in each repetition.

The RVL concentration was defined as the point at which the initially clear oily phase became turbid due to active saturation. Photographs of the oily phase were taken at each step. All procedures were performed in triplicate.

### 2.8 RVL Incorporation and Formulation Scale-Up

A 5% RVL solution in ethanol (EtOH) was prepared, and a determined amount was added to the oil phase of the formulations. After lipid melting, the oil phase was magnetically stirred before receiving the aqueous phase to ensure complete homogenization of the active with the other components. Due to the small amount of EtOH added, it is assumed that it evaporated rapidly and did not interfere with nanoparticle formation. The formulations prepared were as follows: without RVL, standard amount of 20 g (NLC-20); with RVL, 20 g (NLC-RVL-20); without RVL, a scaled-up amount of 200 g (NLC-200); and with RVL, scaled-up (NLC-RVL-200). After preparation, all formulations were stored, as described in Section 2.9.

Formulations were evaluated by DLS, and the data were statistically analyzed by a two-factor factorial ANOVA with interaction (2^2^), considering production scale and the presence of the active as factors. The effects of scale and RVL presence on diameter, PDI, and ZP were assessed. A significance level of 95% was adopted, and when significant differences were detected, pairwise comparisons were performed using Tukey’s test.

### 2.9 Final Stability Assay

The formulations were stored at three different temperatures: 8 °C, room temperature, and 40 °C. Those containing RVL were wrapped in aluminum foil to protect them from light exposure. Hydrodynamic diameter, PDI, and ZP were measured by DLS at 0 and 90 days after preparation.

Chemical stability was evaluated by using UV–vis spectrophotometry (306 nm) to determine the RVL content in the formulations. Samples were diluted in EtOH (1:100), and RVL concentration was calculated based on a previously constructed calibration curve (Figure S1A). Formulations without RVL, analyzed under the same conditions, were used as blanks for the RVL-containing formulations.

### 2.10 Characterization of the NLC Formulation

#### 2.10.1 Laser Diffraction

The formulations were analyzed for PSD using laser diffraction (Cilas 1190 Particle Size Analyzer, WI, USA). This technique allows the evaluation of particles ranging from 0.04 to 2500 μm, using Mie light scattering theory to calculate particle size parameters. The parameters obtained were: percentage of particles with a diameter ≤500 nm (% *p* ≤ 500 nm), mean diameter (μm) measured by passing volume, SPAM, and UR.

SPAM was calculated using the formula ((*d*90 – *d*10)/*d*50), where *d*10, *d*50, and *d*90 correspond to the diameters below which 10%, 50%, and 90% of the particle population (by passing volume) are found, respectively. UR was calculated as (mean diameter by passing volume)/(mean diameter by number). Passing volume considers the volume occupied by a particle population relative to the total particle volume analyzed, whereas passing number measures the frequency of particles within a specific size range, indicating the most common particle size by number. A discrepancy between volume- and number-based distributions suggests a broad particle size range, indicating a heterogeneous distribution, i.e., a nonuniform system. Therefore, low SPAM and UR values indicate system uniformity, while high values suggest the opposite, which is not expected.^15^

#### 2.10.2 Dynamic Light Scattering

DLS was employed to determine the hydrodynamic diameter, ZP, and PDI by using a Zetasizer PRO (Malvern Panalytical, UK). For size and PDI measurements, formulations were diluted in water (1:10,000) and analyzed in a polystyrene cuvette (model DTS0012). For ZP determination, formulations were diluted in water (1:100) and measured by using a folded capillary zeta cuvette (model DTS1070).

#### 2.10.3 In Vitro Release Assay

The release studies were conducted using Franz diffusion cells equipped with a cellulose dialysis membrane (10 kDa). In the donor compartment, 1 mL of each formulation was applied, and 0.5 mL aliquots were collected at the following time points: 0, 0.5, 1, 2, 4, 6, 8, 10, 22, and 24 h. Each aliquot was diluted in 1 mL of the receptor solution consisting of PBS (pH 7.4) supplemented with 0.5% w/v Tween 80. The samples were analyzed by UV–Vis spectrophotometry at 306 nm, and the RVL concentration was calculated based on a previously constructed calibration curve (Figure S1B). The receptor compartment contained approximately 5 mL of the aforementioned solution. All experiments were carried out with the receptor phase maintained at 32 ± 1 °C throughout the study. Formulations with and without RVL at the previously standardized concentration as well as a hydroalcoholic solution (1:1) containing RVL at the same concentrations were evaluated.

#### 2.10.4 Encapsulation Efficiency and Loading Efficiency

Encapsulation efficiency (EE %) corresponds to the amount of RVL encapsulated in the lipid nanoparticles relative to the total amount initially added to the formulation. For this purpose, 1 mL of the formulation was subjected to ultracentrifugation (5000 rpm for 90 min) using Amicon tubes with a 10 kDa cutoff. The amount of unencapsulated bioactive material (filtrate) was diluted in ethanol and quantified by UV–vis spectrophotometry (306 nm). The EE % was calculated by using the following formula

$$EE\;\%=100-\left(\frac{\left[RVL\right]filtrate}{250\cdot total\;recovered\;RVL}\times 100\right)$$

where [RVL] = RVL concentration (μg/mL), 250 = theoretical initial RVL concentration (μg/mL), and total recovered RVL = ratio of actual to theoretical initial RVL concentration.

Loading efficiency (LE %) was calculated using the following formula

$$LE\;\%=\left(\frac{\left[EE\;\%\right]\cdot RVL}{LIP}\right)$$

where *E* % = encapsulation efficiency (%), RVL = amount of RVL added to 100 g of formulation (g), LIP = amount of lipid added to 100 g of formulation (g).

#### 2.10.5 Differential Scanning Calorimetry and Thermogravimetry

Initially, samples of the formulations with and without RVL (NLC-RVL and NLC-blank, respectively) and their isolated raw materials were frozen at −20 °C and subsequently lyophilized using an L108 Lyophilizer (Liotop) for 48 h (44 μHg, −57 °C). The differential scanning calorimetry (DSC)/thermogravimetry (TG) analyses were performed using a simultaneous thermal analyzer DSC/TGA discovery SDT 650 (TA Instruments, Delaware, USA), under a synthetic air atmosphere (50 mL min^–1^), with a heating rate of 10 °C min^–1^, and within a temperature range of 10 to 650 °C. The experiments were conducted in 110 μL platinum crucibles, with open lids, employing sample masses between 7 and 15 mg. Both formulations and the solid and liquid constituents of the systems were analyzed.

The parameters evaluated included mass variation (Δ*w*, %), extrapolated onset degradation temperature (*T*_onset_, °C), DSC peak temperature (*T*_pico_ DSC, °C), and DTG peak temperature (*T*_pico_ DTG, °C). Data processing was carried out using the TA Instruments Universal Analysis 200 (Trios) software, available from TA Instruments.

#### 2.10.6 Fourier Transform Infrared Spectroscopy

Lyophilized samples of the formulations (NLC-blank and NLC-RVL) and their isolated raw materials were used for this analysis. Fourier transform infrared (FTIR) spectra were recorded using an Agilent Cary 630 FTIR spectrometer over a wavelength range of 4000–400 cm^–1^, employing an attenuated total reflectance accessory.^16^

#### 2.10.7 Transmission Electron Microscopy

Transmission electron microscopy (TEM) was performed using a JEM 2100 electron microscope at the Analytical Center of the Institute of Chemistry, University of São Paulo (USP), operating at 200 kV. Copper grids (Pelco, Ted Pella, USA) with 300 mesh and dimensions of 77.5 × 41.0 × 9.0 mm, coated with a carbon film, were used. The NLC-RVL sample was diluted in water (1:10,000 v/v), stained with 1% phosphotungstic acid (Sigma-Aldrich), and allowed to dry at room temperature.^17,18^

### 2.11 Statistical Analysis

All experiments were performed at least in triplicate, with some conducted in quintuplicate. Statistical analysis was carried out using Minitab software, version 18 (Minitab Inc., USA). Mean values were analyzed by one-way ANOVA and when significant differences were detected, the Tukey posthoc test was applied. DoE creation, data analysis via RSM, and formulation optimization were performed using the same software as well as the generation of Pareto charts and contour plots. A significance level of 5% was adopted for all of the analyses.

## 3 Results and Discussion

### 3.1 Selection of Solid Lipid and Surfactant

The first phase of this study was essential for selecting the solid lipid and surfactant to be employed in the subsequent stage. The appropriate choice of both components is a key factor in the development of NLCs, as it is crucial for the solubility of the active ingredients to be incorporated, tissue permeability, and particle stability—a property related to the particle surface charge that can influence not only size stability,^19^ but also the initial size, encapsulation efficiency, and other physicochemical properties.^20^

The spontaneous emulsification process, also known as water titration, is commonly used to produce nanoemulsions using only magnetic stirring, without the need for heating or temperature control during the process.^21,22^ However, for the production of SLN and NLCs using this method, heating is required due to the need for solid lipid melting. Previous studies have reported that such nanoparticles were prepared at temperatures ranging from 70 to 85 °C using the aforementioned method.^23,24^ In one study, the authors applied the method at 70 °C for both the aqueous and oily phases to produce NLCs,^23^ whereas in another study, the authors employed spontaneous emulsification with the oily phase heated to 85 °C, which was then added to the aqueous phase and subsequently cooled in an ice bath.^24^

Although these previous studies share similarities with the present work—namely, the use of the same general method and the production of SLN—they differ in the preparation conditions, thereby highlighting the innovative aspect of our approach. To investigate the impact of different phase temperatures and the type of addition (water or oil titration), we modified the technique by primarily increasing the temperature of the aqueous phase and heating it beyond that of the oil phase.

Using this adapted process, four formulations with suitable particle sizes were obtained: F1, F2, F3, and F6 (Table 1). ANOVA revealed significant differences among formulations (*p* < 0.05). For the parameters % *p* ≤ 500 nm and SPAM, Tukey’s test indicated differences between all formulations (*p* < 0.05), grouping them into six distinct clusters. In contrast, no significant difference was found between F3 and F1 for mean diameter (*p* = 1.00) or between F2 and F1 for RU (*p* = 0.998).

**Table 1:** PSD Obtained by Laser Diffraction with Variations in the Type of Surfactant and Solid Lipid Used^a^

evaluated parameters	F1	F2	F3	F4	F5	F6
surfactant	O20	C20	CORH40	O20	C20	CORH40
solid lipid	CA	CA	CA	GS	GS	GS
% *p* ≤ 500 nm ± SD	99.76 ± 0.04	100 ± 0.0	96.25 ± 0.01	16.74 ± 0.03	34.13 ± 0.05	95.44 ± 0.11
average diameter (μm) ± SD	0.20 ± 0.00	0.19 ± 0.00	0.20 ± 0.00	5.90 ± 0.01	4.75 ± 0.01	0.21 ± 0.00
SPAM ± SD	1.58 ± 0.00	1.61 ± 0.00	1.94 ± 0.00	2.91 ± 0.00	3.68 ± 0.01	2.13 ± 0.00
UR ± SD	4.67 ± 0.58	4.75 ± 0.00	2.50 ± 0.00	58.97 ± 0.06	118.83 ± 0.14	3.50 ± 0.00

a % *p* ≤ 500 nm ± SD: percentage of particles with diameter less than or equal to 500 nm ± standard deviation; SPAM ± SD: dispersity ± standard deviation; UR ± SD: uniformity ratio ±standard deviation; F0: initial formulation; FR: repeated formulation. O20: Oleth-20; C20: Ceteareth-20; CORH40: PEG-40 hydrogenated castor oil; CA: cetearyl alcohol; GS: glyceryl stearate.

Initially, formulations F1, F2, F3, and F6, which had shown good performance, remained stable for 2 days. Among them, F1 exhibited the greatest variation; however, its size and uniformity remained within the defined parameters (Figure S2). In contrast, formulations F4 and F5 showed poorer stability performance, with significantly higher mean diameter and RU values (Figure S2C,F).

Formulations F1, F2, F3, and F6 were then prepared again under the same initial conditions to assess the reproducibility. Among these, F2 and F6 displayed the smallest variations in parameters compared to their initially prepared counterparts (Table S1).

Based on the reproducibility results of the best-performing formulations, F2 and F6 were considered the most satisfactory. F2 contains the solid lipid CA and the surfactant Ceteareth-20, while F6 contains GS and the surfactant PEG-40 hydrogenated castor oil (CORH40).

Similarly, a previous study reported the use of Ceteareth-20 and CORH40 together to develop nanoemulsions via the phase inversion technique at 80 °C, which yielded droplets smaller than 200 nm.^25^ Therefore, we will investigate the combined use of these two surfactants in our next experiment. As the intention is to work with only one solid lipid, CA was selected for the subsequent stages.

### 3.2 Factorial Study for Formulation-Related Variables

#### 3.2.1 Effect of Independent Variables on PSD

The PSD values obtained by the DoE are presented in Table 2. The formulations exhibited % *p* ≤ 500 nm ranging from 34 to 100%, average diameters between 180 and 5010 nm, SPAM values between 1.56 and 9.12, and UR values between 2.38 and 125.25. Among all tested formulations, R3F1 and R9F3 were the only ones that did not meet the target PSD criteria for all of the evaluated parameters.

**Table 2:** DoE and PSD for Formulations Prepared with Variations in Composition

run order	formulation code	[surfactant]	CORH40 ratio^b^	[lipid phase]^c^	% *p* ≤ 500 nm ± SD	average diameter (μm) ± SD	SPAM ±SD	RU ± SD
1	4	7	100	4	100 ± 0.00	0.18 ± 0.00	1.65 ± 0.00	3.9 ± 0.52
2	15	5	50	4	95.94 ± 0.10	0.20 ± 0.00	2.04 ± 0.04	3.17 ± 0.27
3^a^	1	3	0	4	34 ± 0.20	5.01 ± 0.04	6 ± 0.00	125.25 ± 0.00
4	9	5	0	2	94.99 ± 0.04	0.21 ± 0.00	2.06 ± 0.00	3.5 ± 0.00
5	10	5	100	2	99.81 ± 0.02	0.20 ± 0.00	1.58 ± 0.00	4.67 ± 0.58
6	8	7	50	6	100 ± 0.00	0.19 ± 0.00	1.56 ± 0.00	4.75 ± 0.00
7	14	5	50	4	100 ± 0.00	0.19 ± 0.00	1.56 ± 0.00	4.75 ± 0.00
8	5	3	50	2	97.18 ± 0.09	0.19 ± 0.00	1.81 ± 0.00	2.38 ± 0.00
9^a^	3	3	100	4	56.01 ± 0.08	1.31 ± 0.01	9.12 ± 0.05	18.67 ± 0.08
10	11	5	0	6	99.94 ± 0.00	0.19 ± 0.00	1.53 ± 0.00	4.75 ± 0.00
11	6	7	50	2	96.88 ± 0.36	0.20 ± 0.01	2.02 ± 0.04	3.28 ± 0.10
12	12	5	100	6	98.91 ± 0.03	0.20 ± 0.00	1.55 ± 0.00	5.00 ± 0.00
13	2	7	0	4	96.35 ± 0.09	0.20 ± 0.00	2.00 ± 0.06	2.90 ± 0.42
14	13	5	50	4	97.87 ± 0.01	0.19 ± 0.00	1.80 ± 0.00	2.38 ± 0.00
15	7	3	50	6	99.18 ± 0.06	0.20 ± 0.00	1.55 ± 0.00	5.00 ± 0.00

a Means that the formulations did not achieve adequate PSD values.

b Means C20 was used to reach 100% of surfactant when [CORH40] was lower than 100%.

c Means lipid phase compounded with CCT/CA (1:1). [Surfactant]: surfactant concentration (% *p*/*p*). [Lipid phase]: lipid phase concentration (% *p*/*p*).

The RSM analysis yielded *R* ^2^ values (%) of 71.72, 72.21, 71.10, and 72.25 for % *p* ≤ 500 nm, average diameter, SPAM, and UR, respectively. For % *p* ≤ 500 nm and SPAM, the most influential factor was [surfactant], followed by the quadratic interaction of [lipid phase], [surfactant], and CORH40 proportion. In contrast, for average diameter and UR, the interaction between [surfactant] and CORH40 proportion, as well as [surfactant] alone, was the primary determinant of producing smaller and more uniform nanoparticles (Figure S3). The quadratic regression model equations, including linear, quadratic, and interaction terms, are provided in the Supporting Information (Table S2).

The contour plots highlight the regions for formulation optimization (Figure 2). For achieving a higher % *p* ≤ 500 nm, optimal conditions include [surfactant] between 5 and 7% and CORH40 proportions between 30 and 80%. When [surfactant] exceeds 5%, the lipid concentration becomes less critical, with a broad range still yielding particles <250 nm—a trend also observed for SPAM. For minimizing UR, it is advantageous to increase [surfactant] in proportion to the C20 percentage. Notably, when % CORH40 is low, higher [surfactant] levels are required to achieve the same effect. These findings indicate that smaller and more uniform particles are obtained when [surfactant] is high, regardless of C20 concentration. Additional combinations of variables and their corresponding responses are detailed in the Supporting Information (Figure S4).

**Figure 2:**
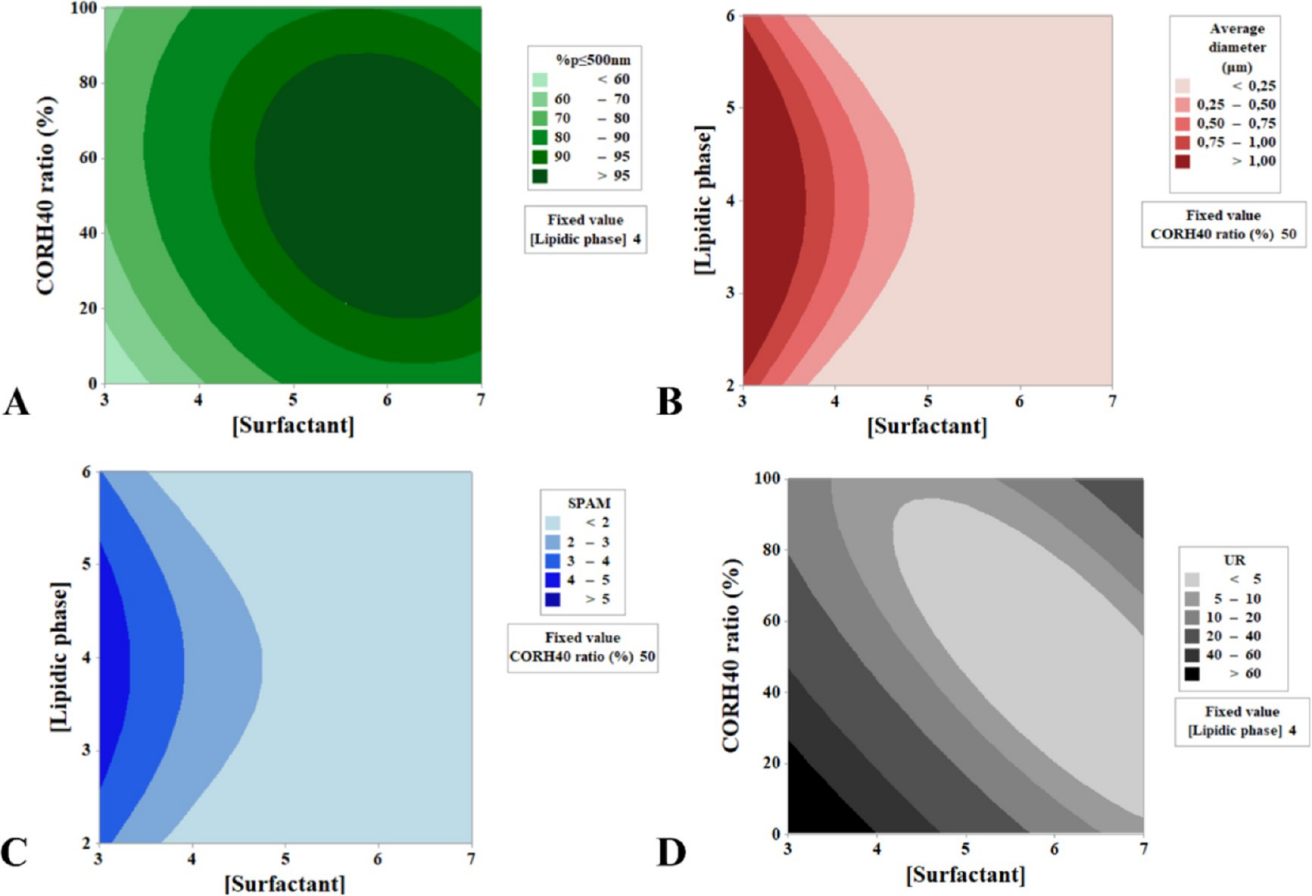
Contour plots generated by RSM for different interactions between formulation composition variables to optimize PSD values. (A) % *p* ≤ 500 nm, (B) average diameter (μm), (C) SPAM, and (D) UR.

#### 3.2.2 Formulation Optimization

Optimization of the formulation composition indicated that 5.9% [surfactant], composed almost entirely of CORH40, combined with a lipid phase concentration of 5.7%, achieved the target PSD parameters with a high desirability value of 0.9976. The predicted and observed results for % *p* ≤ 500 nm were 98% and 95%, respectively. For the average diameter, the predicted value was 0.19 μm, and the observed value was 0.20 μm. For SPAM, the predicted and observed values were 1.67 and 2.00, respectively, while for UR, they were 4.55 and 2.54, respectively. These results demonstrate that formulation optimization was successfully achieved through a factorial design study.

The optimized formulation was subsequently evaluated for preliminary stability over a 15 day period. Regarding PSD, a slight increase in particle size was observed: the population ≤500 nm decreased from 95% to approximately 80%, while the average diameter increased from 0.20 to 0.55 μm, remaining within the nanometric range. Consequently, the reduction in the proportion of smaller particles led to greater system heterogeneity, as reflected in increased SPAM and UR values. Phase separation began gradually on the second day, coinciding with the increase in particle size, and progressed in a similar manner thereafter (Figure 3).

**Figure 3:**
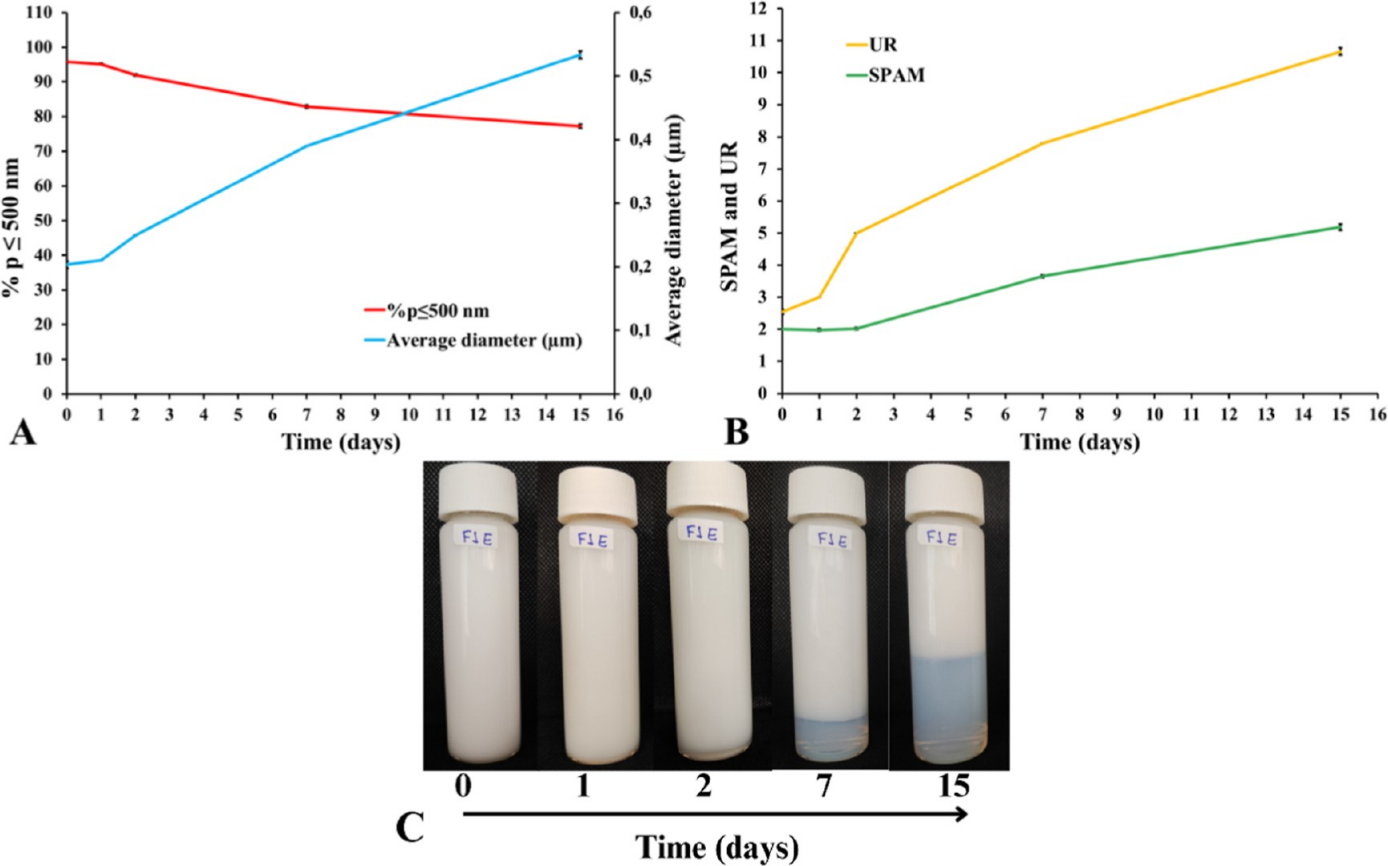
Preliminary stability of optimized formulation (A) % *p* ≤ 500 nm and average diameter (μm), (B) SPAM and UR, and (C) aspect and phase separation.

### 3.3 Factorial Study of Process-Related Variables

#### 3.3.1 Effect of Independent Variables on Particle Size and Uniformity

The factorial DoE investigating variables associated with the method yielded 26 formulations, of which 10 exhibited PSD values within the predefined limits. The % *p* ≤ 500 nm ranged from 0.23 to 95.96%, the mean diameter from 0.20 to 27.7 μm, SPAM from 1.21 to 161.4, and UR from 2.62 to 647.38 (Table 3).

**Table 3:** DoE and PSD of Formulations Obtained Using Process Variables

formulation (run + code)	oily phase temperature (°C)	aqueous phase temperature (°C)	stirring speed (rpm)	order of the addition of phases	% *p* ≤ 500 nm	average diameter (μm)	SPAM	UR
R1F13	63	85	550	aqueous over oily	26.59	27.70	5.10	647.38
R2F14	58	85	400	oily over aqueous	2.11	24.30	2.58	366.29
R3F26	63	85	550	oily over aqueous	0.89	19.19	1.22	122.57
R4F19	68	80	550	oily over aqueous	0.23	19.56	1.21	25.74
R5F12^a^	63	90	700	aqueous over oily	95.93	0.20	2.00	2.86
R6F2^a^	68	85	400	aqueous over oily	95.96	0.20	2.00	2.62
R7F17	68	85	700	oily over aqueous	19.82	11.35	2.63	265.12
R8F24	63	90	400	oily over aqueous	13.12	7.84	2.26	78.37
R9F10^a^	63	80	700	aqueous over oily	95.65	0.21	2.08	3.50
R10F25	63	90	700	oily over aqueous	1.73	17.61	1.28	203.08
R11F5	58	80	550	aqueous over oily	62.93	12.15	161.40	303.67
R12F15	68	85	400	oily over aqueous	5.20	11.09	1.92	184.78
R13F7^a^	58	90	550	aqueous over oily	95.62	0.21	2.10	3.50
R14F21	68	90	550	oily over aqueous	89.47	0.31	2.19	5.87
R15F20^a^	58	90	550	oily over aqueous	94.74	0.21	2.10	3.50
R16F8^a^	68	90	550	aqueous over oily	95.47	0.21	2.13	3.50
R17F9^a^	63	80	400	aqueous over oily	94.92	0.21	2.06	3.50
R18F22	63	80	400	oily over aqueous	13.95	5.83	2.62	58.30
R19F16^a^	58	85	700	oily over aqueous	95.19	0.21	2.06	3.97
R20F4	68	85	700	aqueous over oily	66.91	2.52	35.17	50.47
R21F23	63	80	700	oily over aqueous	68.15	2.18	31.20	43.53
R22F11^a^	63	90	400	aqueous over oily	94.60	0.21	2.12	4.20
R23F18	58	80	550	oily over aqueous	14.94	6.16	2.72	55.97
R24F1^a^	58	85	400	aqueous over oily	95.17	0.21	2.06	3.50
R25F6	68	80	550	aqueous over oily	69.25	2.48	36.31	49.60
R26F3	58	85	700	aqueous over oily	65.20	3.62	38.86	90.58

a Indicate NLC formulations that met the predefined ideal parameters.

According to RSM analysis, the coefficient of determination (*R* ^2^, %) for each PSD parameter was as follows: % *p* ≤ 500 nm, 92.98; average diameter—87.54, SPAM—91.87, and UR—76.85. The most significant independent variable across all PSD parameters was the order of phase addition, either alone or in interaction with the oil-phase temperature or stirring speed (Figure S5). The addition of the aqueous phase over the oil phase was found to be the most suitable approach for producing nanoparticles, and this was consistently observed in all of the best-performing formulations (marked with *, Table 3). Overall, nearly all factors and their interactions were statistically significant for particle size when this method was applied. The aqueous-phase temperature emerged as the most influential variable for SPAM but was not significant for UR—a somewhat unexpected finding given that both parameters assess uniformity. The quadratic regression model equations are provided in the Supporting Information (Table S3).

Contour plots illustrate the optimal process conditions for producing smaller and more uniform particles when the aqueous phase is added over the oil phase (Figure 4). For higher % *p* ≤ 500 nm, the optimal conditions are stirring speed of 400–450 rpm, oil-phase temperature of 60–65 °C, and aqueous-phase temperature of 88–90 °C. For lower SPAM values, stirring within the same range (400–450 rpm) is recommended, allowing a broader temperature range for both phases. However, at a stirring speed of 550 rpm, an aqueous-phase temperature above 88 °C is preferred. For lower UR values, the aqueous-phase temperature may be reduced to approximately 85 °C if the oil-phase temperature is maintained at 65 °C. Additional variable combinations for the responses are provided in the Supporting Information (Figure S6).

**Figure 4:**
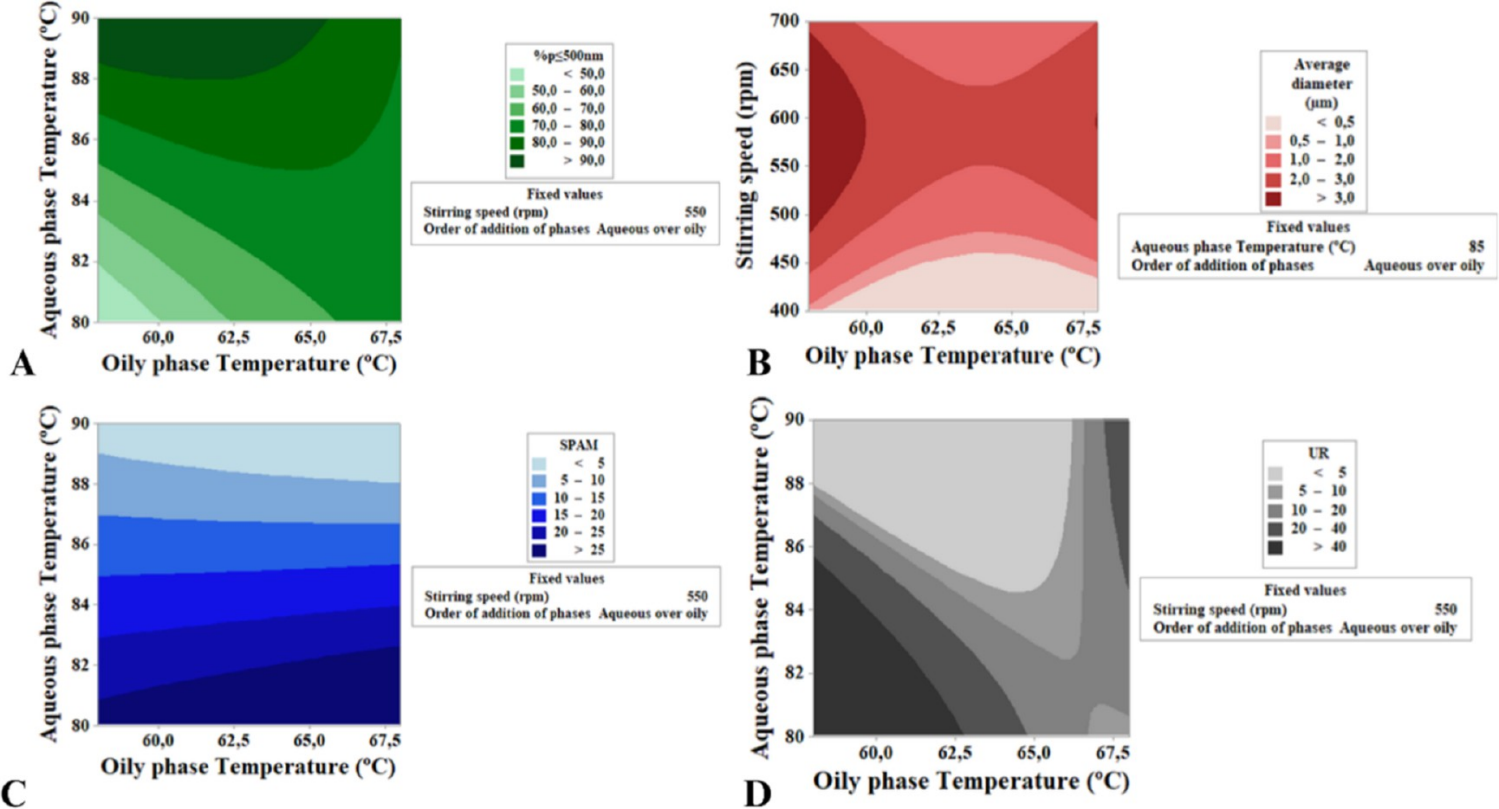
Contour plots generated by RSM for different interactions among the process variables. (A) % *p* ≤ 500 nm; (B) average diameter (μm); (C) SPAM; (D) UR.

A previous study also reported the development of NLC using an emulsification method without organic solvents or specialized equipment. In that work, both phases were heated to 85 °C, resulting in NLC with a mean size of 90 nm and a PDI of 0.147, demonstrating that higher temperatures favor nanoparticle formation, while subsequent cooling is necessary for solidification.^26^

#### 3.3.2 Process Optimization

Process optimization identified the most favorable conditions for producing nanoparticles using the low-energy approach, achieving a desirability of 0.9059. The optimized conditions were: oily phase temperature = 64 °C, aqueous phase temperature = 90 °C, stirring speed = 642 rpm, and order of phase addition = aqueous over oily. The predicted and observed values for % *p* ≤ 500 nm were 93 and 95, respectively; for mean diameter, 0.28 μm (predicted) and 0.20 μm (observed); for SPAM, 2.5 μm (predicted) and 2 μm (observed); and for UR, 3.19 μm (predicted) and 2.5 μm (observed).

The optimized formulation (OFE) and its scaled-up version (SFE) exhibited similar stability profiles and PSD characteristics, both showing the presence of phase separation. Statistical analysis revealed significant differences in all pairwise comparisons. Nevertheless, both formulations began the stability study with approximately 95% *p* ≤ 500 nm and ended with about 80%, with mean diameters increasing from 0.2 to 0.5 μm. SPAM values rose from ∼2 to ∼5, and UR from 2.5 to 10 between 0 and 15 days after preparation, respectively, indicating that the process is robust and not significantly affected by production scale (Figure 5). However, the need for formulation improvement to extend the stability period remains evident.

**Figure 5:**
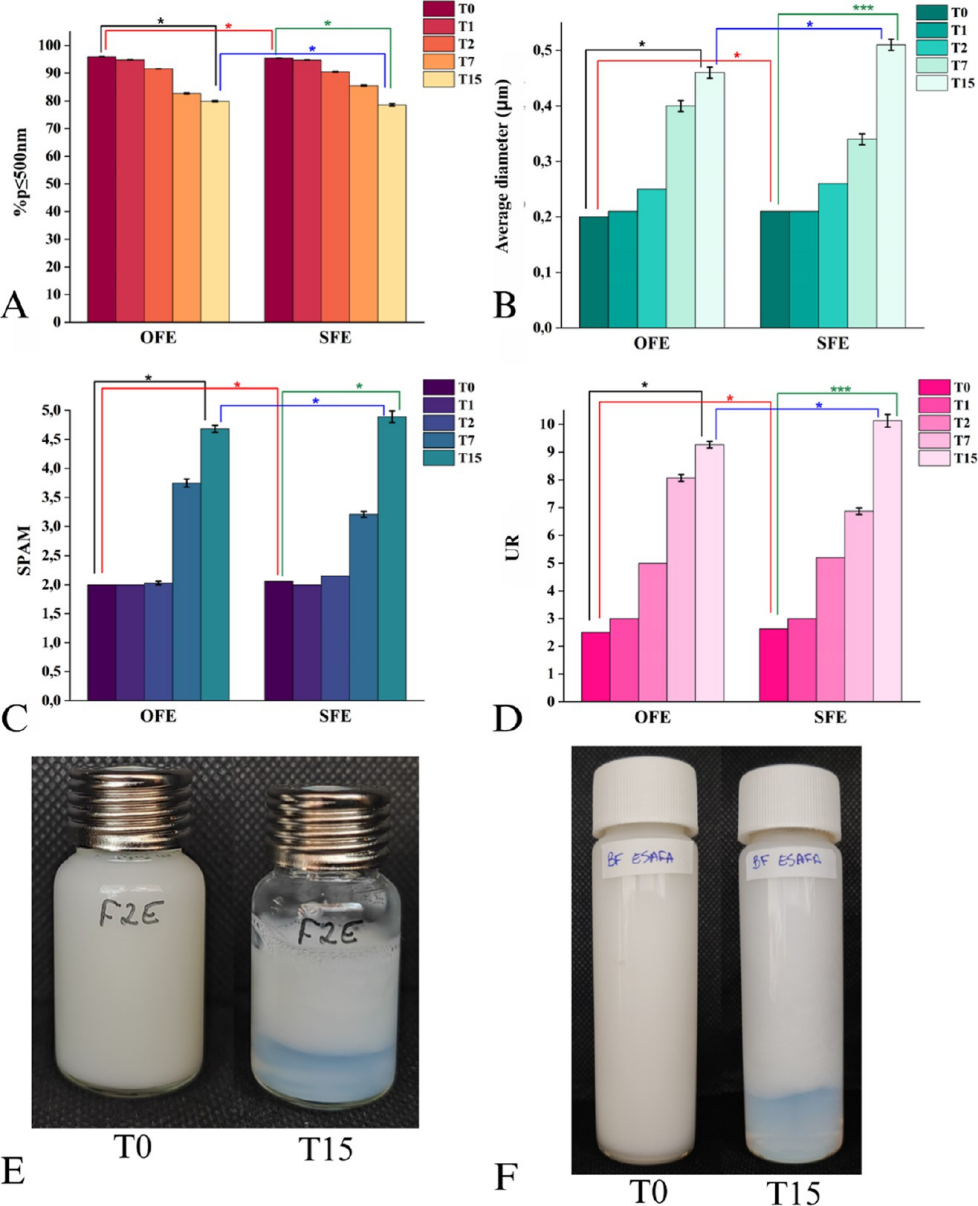
Stability of the optimized and scaled-up NLC formulation, * means *p* < 0.05; OFE: optimized formulation, SFE: scaled-up formulation, (A) % *p* ≤ 500 nm, (B) average diameter (μm), (C) SPAM (D) UR, (E) OFE aspect, and (F) SFE aspect; T0: preparation day, T15: 15 days after preparation.

### 3.4 Stability Improvement

#### 3.4.1 Surface Ionic Charge Addition

Electrostatic stabilization is a mechanism that prevents particle aggregation in a colloidal system. Particles carrying a positive or negative charge tend to repel each other; therefore, ZP values below −30 mV or above +30 mV indicate stability due to electrostatic repulsion, whereas values close to zero suggest opposite surface charges, facilitating particle aggregation and possible coalescence.^27^

DLS analysis of the formulations revealed particle sizes ranging from 87 to 110 nm, PDI values between 0.22 and 0.28, and ZP values between −58 and 57 mV (Table 4).

**Table 4:** DLS Results for Formulations Containing Ionic Surfactants at Different Concentrations^a^

formulation code	surfactant + concentration (%)	size (nm)	PDI	ZP (mV)
F1	SLS 0.05	99.43 ± 1.28	0.262 ± 0.02	–47.93 ± 8.57
F2	SLS 0.1	103.17 ± 0.80	0.247 ± 0.01	–32.33 ± 9.68
F3	SLS 0.2	107.53 ± 1.67	0.255 ± 0	–58.88 ± 6.24
F4	CTAC 0.05	98.28 ± 0.97	0.256 ± 0.01	31.02 ± 4.1
F5	CTAC 0.1	106.67 ± 1.88	0.288 ± 0	47.55 ± 5.76
F6	CTAC 0.2	110.07 ± 1.27	0.255 ± 0.01	57.56 ± 5.74
F7		87.64 ± 0.47	0.224 ± 0.01	–3.41 ± 0

a F1–F3: SLS; F4–F6: CTAC; F7: control formulation without charge-donating surfactants.

According to ANOVA, the formulations differed significantly in particle size, PDI, and ZP (*p* < 0.05). Tukey’s test indicated that F7 (control) had the smallest particle size compared to all other formulations, suggesting that the addition of SLS or CTAC did not favor nanoparticle formation. Both F7 and F2 showed the lowest PDI values, without a significant difference (*p* > 0.05). As expected, the cationic surfactant CTAC imparted positive charges to nanoparticles, reaching +57 mV at the highest concentration (F6), while the anionic SLS produced negative charges, with the lowest value of −57 mV for F3. The control formulation (F7) remained near neutral due to the nonionic surfactant CORH40.^28^

Preliminary stability over 14 days (Figure 6A) showed that all formulations exhibited changes in particle size and PDI, with F7 presenting the largest variation (>1000%), losing its nanometric character and reaching a PDI of 1, indicative of heterogeneity. F1 and F2 showed lower, though still considerable, variations (>190%), with no significant size difference at day 14 (*p* > 0.05). Regarding PDI, F1 had a significantly lower mean compared to F7 (*p* < 0.05). ZP remained largely unchanged during the study (Figure 6B).

**Figure 6:**
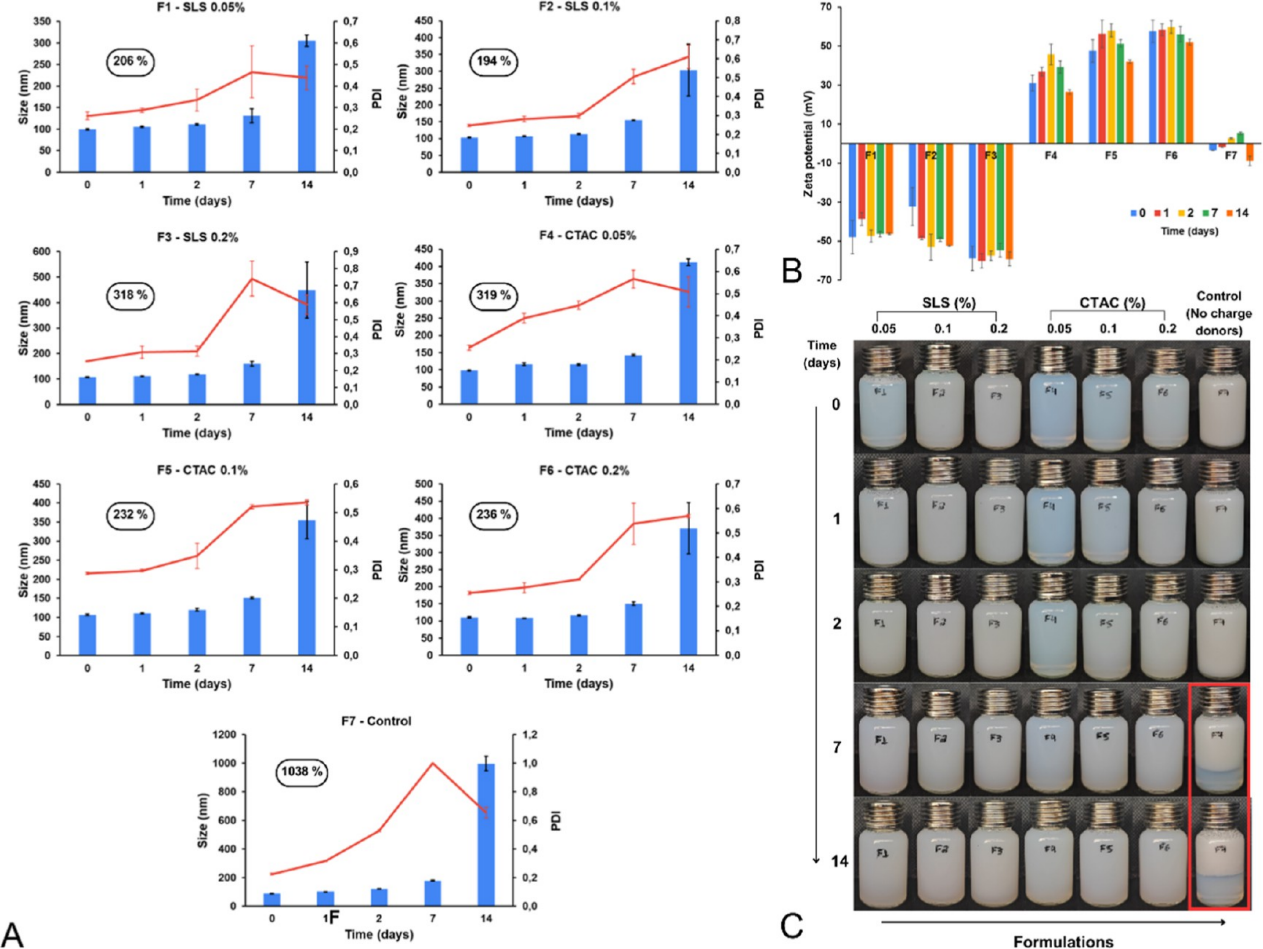
Preliminary stability of NLC formulations with added ionic surfactants: (A) Particle size and PDI, (B) ZP, and (C) visual appearance of the formulations.

Formulations containing ionic surfactants also displayed distinct visual characteristics: at day 0, F1, F4, and F5 appeared translucent with a bluish hue, typical of nanosized systems (100–1000 nm).^29^ Phase separation was observed in the control from day 7, demonstrating that the addition of either SLS or CTAC effectively enhanced the physical stability across all tested concentrations (Figure 6C).

Based on the results, formulation F1 was selected for subsequent studies due to its combination of favorable properties: low initial particle size (comparable to F4), moderate size variation during stability (206%), one of the lowest mean sizes at T14 (along with F2), lowest PDI at T14, and a ZP near −50 mV at the end of the study.

A study reported similar particle size (127 nm) and ZP (−48.9 mV) using 0.5% SLS in optimized fexofenadine-loaded CLNs.^30^ Despite differences in overall formulation composition, these findings support the effectiveness of SLS in producing stable nanoparticles.

#### 3.4.2 Modification of the Dispersing Polymer

Steric stabilization prevents particle aggregation by coating nanoparticles with hydrophilic polymers, enhancing stability even when ZP is low and electrostatic stabilization is limited.^27,31^

PVP, a nonionic, hydrophilic, and nontoxic polymer, is widely used as a steric stabilizer and surface modifier for nanocarriers.^32,33^ P188, a nonionic amphiphilic copolymer, is also employed in pharmaceutical and cosmetic formulations^34^ and has demonstrated SLN stabilization for 180 days at 2.5% in the aqueous phase,^35^ which justified its use in this study.

The formulations showed initial particle sizes of 99, 107.8, and 111.1 nm for F1, F2, and F3, respectively (*p* < 0.05), with PDI values of 0.271, 0.314, and 0.287 (*p* = 0.299) and zeta potentials of −43.6, −41, and −50.2 mV (*p* = 0.106).

During stability studies, particle size increased from day 7 onward, with no significant difference at day 14 (*p* = 0.340) (Figure 7A). PDI showed a significant difference (*p* = 0.031), with F3 not differing from F1, and F1 not differing from F2, indicating that the difference lies between F3 and F2 (P188 vs 10% PVP). Polymer substitution did not impact final PDI, as F2 and F3 were not significantly different from F1; F2 exhibited the lowest PDI at day 14 (0.54), similar to F1 (0.55) (Figure 7B).

**Figure 7:**
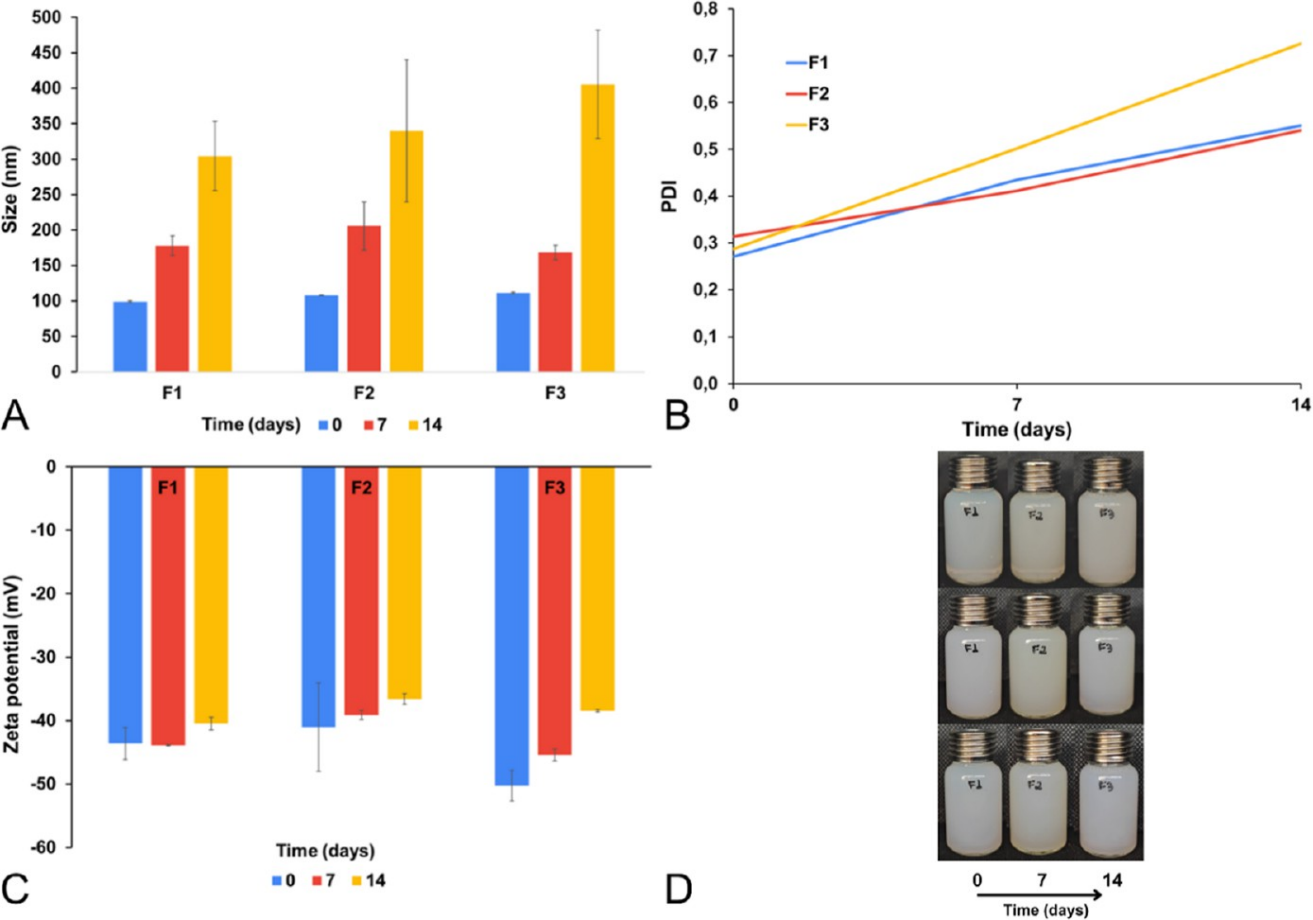
Preliminary stability of NLC formulations with modified dispersion Polymer. (A) Particle size, (B) PDI, (C) ZP, (D) appearance of formulations. F1: PVP 2%—control (unmodified), F2: PVP 10%, F3: P188 2.5% + PVP 2%.

During the stability study, a decrease in ZP was observed in all formulations, most pronounced in F3, which changed from −50 to −38 mV at T14 (Figure 7C). ANOVA revealed a significant difference in ZP at T14 (*p* = 0.002), with F1 showing the lowest value (−40 mV). These results indicate that polymer substitution was not advantageous, as it reduced ZP, making it less negative and potentially compromising colloidal stability. However, phase separation was absent in all three formulations (Figure 7D). Therefore, the formulation from the previous step was retained without modifications to the dispersion polymer.

### 3.5 RVL Solubility Assay

The solubility of the active in the release assay’s receptor medium is critical to maintain sink conditions, which directly affect the diffusion rate of the compound across the membrane and its release from the nanoparticle.^36^ Different concentrations of Tween 80 and/or ethanol in PBS were tested to identify the solution providing the highest RVL solubility for use as the receptor medium. Each tube contained 300 μg/mL of RVL.

Results (Figure 8A) showed that PBS solutions containing only ethanol tended to darken after 48 h, suggesting possible degradation of RVL in higher-alcohol media, despite ethanol being a good solubilizer for RVL.^37^ RVL’s low stability under various conditions, particularly in alkaline solutions (pH 8–10) due to hydrolysis, and under UV exposure, causing trans-to-cis isomerization, has been widely reported.^38^ PBS solutions with 0.5% and 1% Tween 80 achieved the highest solubilized RVL concentrations, differing by only 6 μg. Therefore, PBS with 0.5% Tween 80, solubilizing 270 μg/mL RVL, was selected as the receptor medium for the release assay.

**Figure 8:**
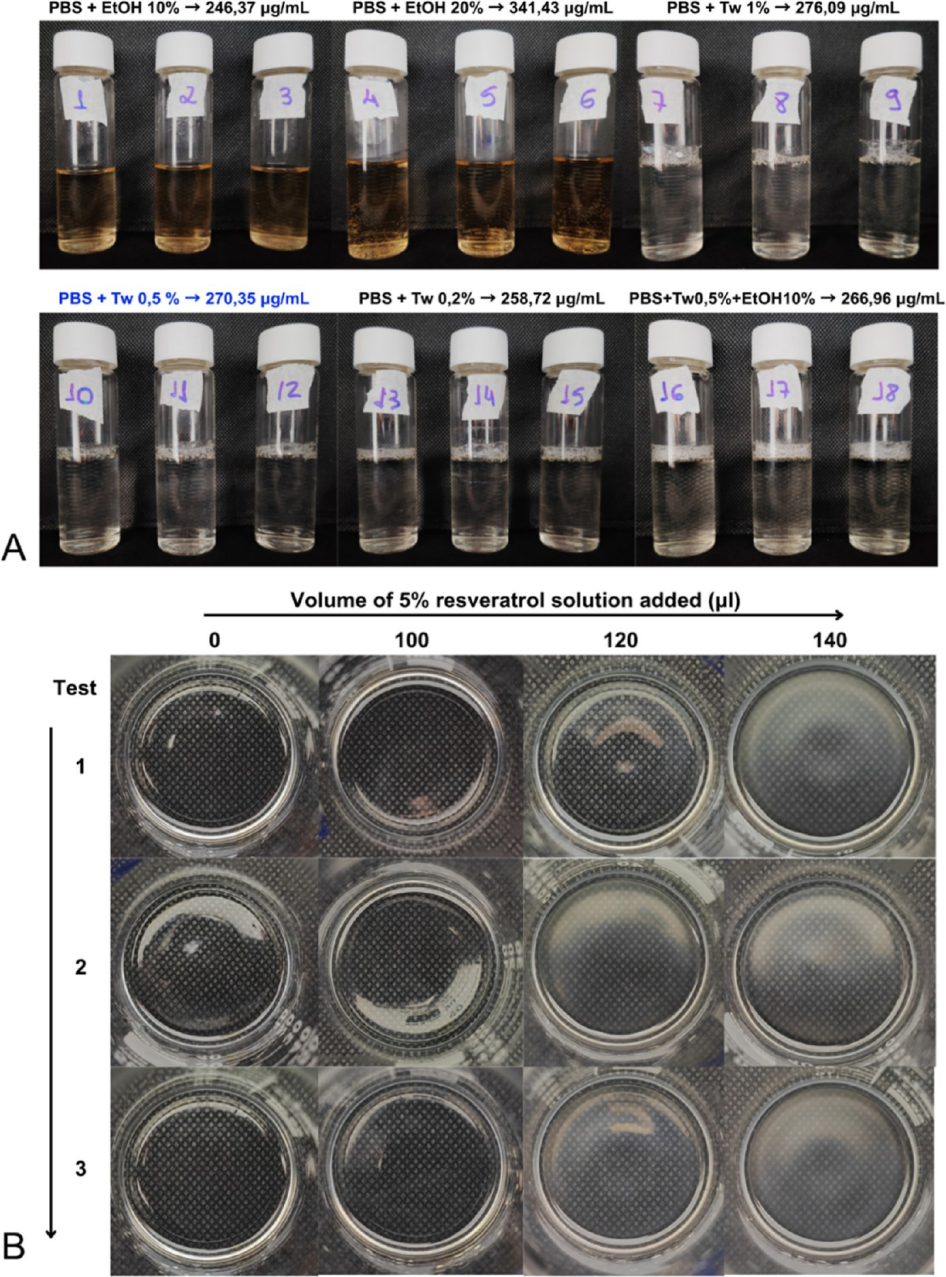
Solubility of RVL in (A) different PBS solutions and (B) the oily phase of the formulation (CA, CCT, and CORH40).

To determine the maximum concentration of RVL to be incorporated into the formulation, a solubility assay was performed in the oily phase (lipid and surfactant mixture) to monitor visual changes. The point of turbidity, indicating saturation, was recorded. After 100 μL of a 5% ethanolic RVL solution was added, no change in appearance was observed compared to the blank oily phase. However, adding an additional 20 μL resulted in turbidity, suggesting saturation of the medium (Figure 8B). Therefore, 100 μL of the RVL solution was established as the maximum amount to be added to 20 g of the oily phase, corresponding to a concentration of 250 μg/mL of RVL.

The compositions of the formulations selected at each stage of development, from the selection of raw materials to the incorporation of RVL, are presented in Table S4.

### 3.6 RVL Incorporation and Formulation Scale-Up

Particle size ranged from 93 to 175 nm, with PDI values between 0.27 and 0.42. As expected, the ZP was negative due to the presence of SLS. The theoretical RVL concentration was 250 μg/mL; however, actual measurements were higher, with NLC-RVL-200 at 21% above the expected value and NLC-RVL-20 at 6.4% above the expected value (Table 5). These discrepancies may result from pipetting variability, analytical interference, or differences in active compound purity.

**Table 5:** Particle Size, PDI, ZP, and RVL Concentration Determined by DLS and Spectrophotometry Analysis^a^

formulation	size (nm) ± SD	PDI ± SD	ZP (mV) ± SD	[RVL] μg/mL
NLC-20	93.92 ± 1.80	0.271 ± 0.01	–28.55 ± 6.76	
NLC-RVL-20	89.97 ± 1.14	0.283 ± 0.01	–37.36 ± 0.59	265.9
NLC-200	202.08 ± 19.86	0.420 ± 0.11	–46.83 ± 2.31	
NLC-RVL-200	175.92 ± 28.05	0.349 ± 0.09	–42.12 ± 2.03	304.7

a [RVL]: resveratrol concentration.

Two-way ANOVA indicated that scale significantly influenced particle size (*p* = 0.0001), with larger particles in the scaled-up formulations (mean 212 nm, Group A) compared to the standard scale (mean 91 nm, Group B). RVL addition alone did not significantly affect particle size (*p* = 0.1354), and no significant interaction between scale and RVL was observed (*p* = 0.1777). However, Tukey’s analysis suggested a minor contribution of RVL in reducing particle size in scaled formulations, though not statistically robust (Table S5).

PDI analysis mirrored the particle size trends. Scale significantly affected PDI (*p* = 0.0052), with higher values in scaled-up formulations, indicating reduced uniformity. RVL had no significant effect (*p* = 0.1729), and the interaction effect was weak (*p* = 0.109), suggesting only a slight tendency of RVL to reduce PDI in scaled formulations (Table S5).

ZP was significantly influenced by scale (*p* = 0.0007), with more negative values in scaled-up formulations, suggesting an increased colloidal stability. RVL alone did not significantly affect ZP (*p* = 0.370), but the interaction between scale and RVL was significant (*p* = 0.014): RVL decreased ZP in standard formulations (from −28 to −37 mV) but had minimal effect in scaled-up formulations (from −46 to −42 mV) (Table S5).

### 3.7 Final Stability Assay

Regarding particle size, the formulations remained stable when stored at 8 °C, while greater instability was observed at 40 °C and room temperature. Formulations stored at 8 °C exhibited minimal size variation throughout the study (Figure 9A). Similarly, PDI values were more stable under refrigeration, remaining close to 0.4 at the end of the study, whereas other storage conditions led to a significant loss of uniformity, with PDI approaching 1 for all formulations (Figure 9B). ZP remained negative (minimum −20 mV) across all storage conditions (Figure 9C). RVL content showed minimal variation at 8 °C; at room temperature, a moderate decrease was observed, while at 40 °C, the largest reduction in RVL concentration occurred (Figure 9D). These results indicate that both physical and chemical stabilities of the formulations—regardless of RVL presence or scale—require storage at 8 °C.

**Figure 9:**
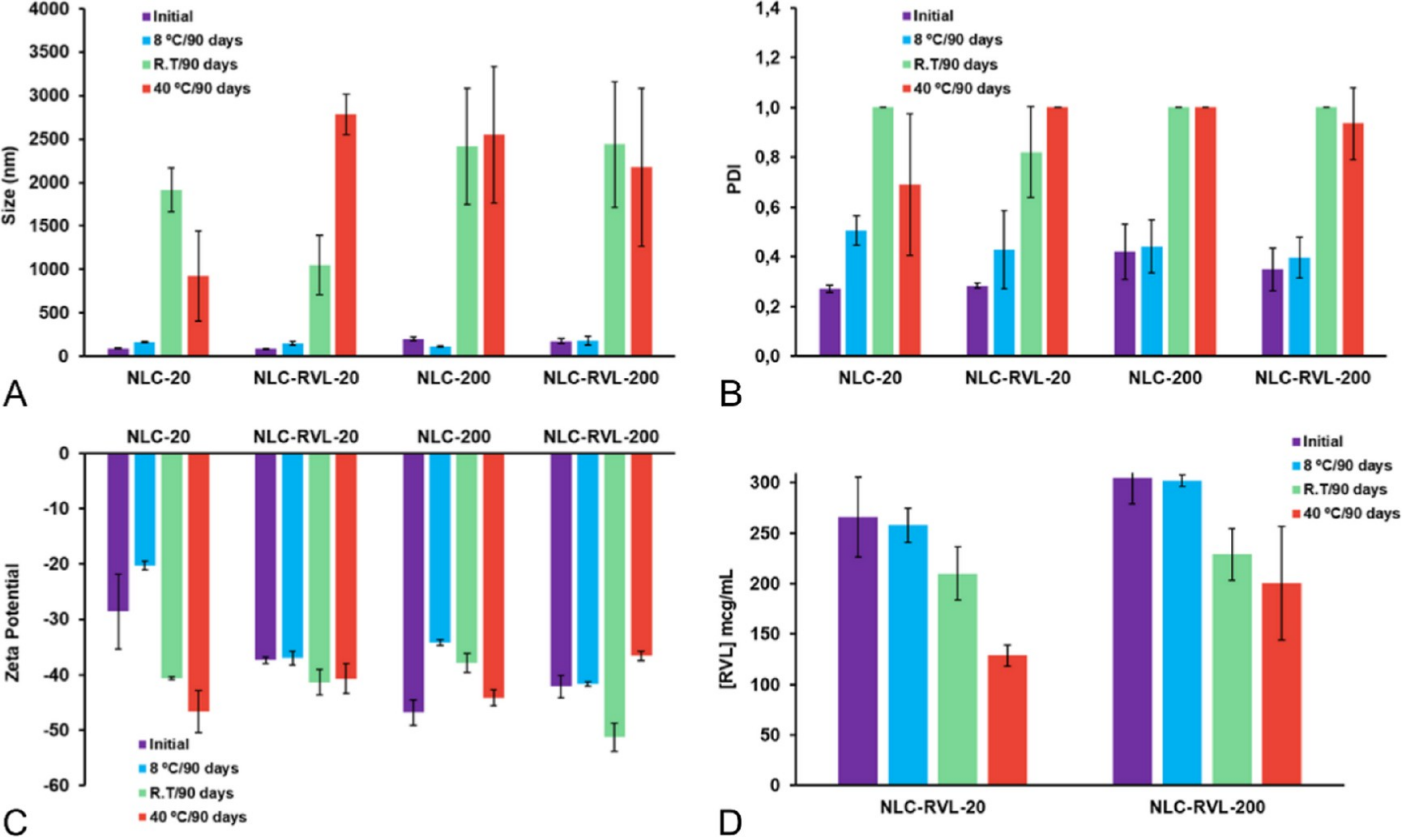
Stability assessment of the formulations over 90 days at different temperatures. (A) Particle size; (B) PDI; (C) ZP; and (D) RVL concentration (μg/mL).

### 3.8 Characterization of the NLC Formulation

#### 3.8.1 Encapsulation Efficiency and Loading Efficiency

Encapsulation efficiency (EE %) was evaluated using tubes equipped with a 10 kDa semipermeable membrane, which retains the nanoparticles while allowing free RVL to pass through. The filtrate containing free RVL was quantified by spectrophotometry, yielding an absorbance of 0.0467 ± 0.009. This corresponded to a low free RVL concentration of 2.34 ± 0.44 μg/mL (0.83 ± 0.16%). Therefore, the encapsulation efficiency was calculated as 99.17 ± 0.16%, demonstrating the process and formulation’s high capacity to effectively incorporate RVL into the nanoparticles. Loading efficiency (LE %) resulted in 0.21%.

#### 3.8.2 In Vitro Release Assay

The optimized RVL-loaded formulation (NLC-RVL) and the RVL hydroalcoholic solution were prepared to achieve a theoretical RVL concentration of 250 μg/mL. The measured concentrations were 266.32 μg/mL for NLC-RVL and 253.94 μg/mL for the solution.

Release kinetics (Figure 10) showed a higher diffusion of RVL through the membrane when solubilized in the hydroalcoholic solution, reaching approximately 60% by the end of the assay. In contrast, the nanostructured formulation exhibited a slower release profile, with only 2% of RVL released by the second hour, 4.5% by the 10th hour, and a total of 8% at the end of the assay. This indicates a prolonged and delayed release, as less than 1% of RVL was released during the first hour.

**Figure 10:**
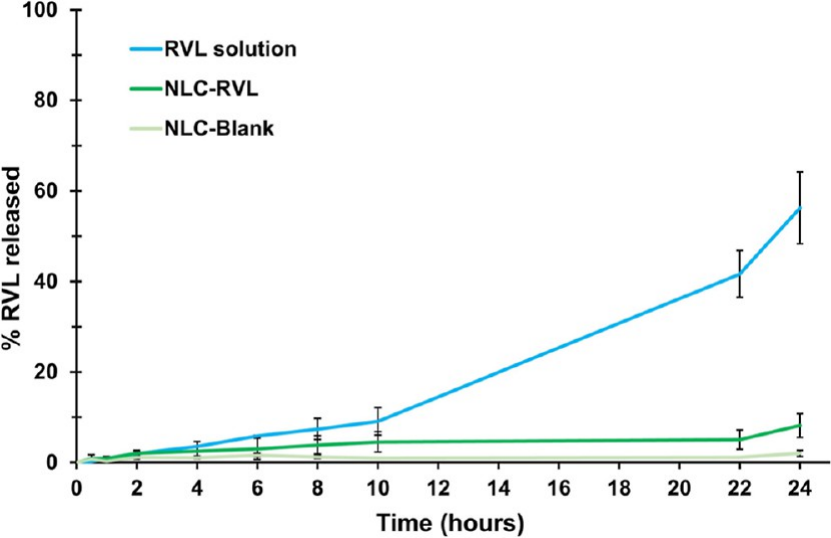
Release kinetics of RVL-loaded formulation and RVL hydroalcoholic solution.

#### 3.8.3 DSC and TG

The raw materials and lyophilized formulations were characterized by TG (Figure 11A) and DSC (Figure 11B,C) aiming to identify the thermal profile and identity pattern of each raw material. Table S6 presents the thermal events, mass loss (Δ*w* %), extrapolated onset degradation temperature (*T*_onset_), DSC peak temperature (*T*_peak_ DSC), and derivative TG peak temperature (*T*_peak_ DTG).

**Figure 11:**
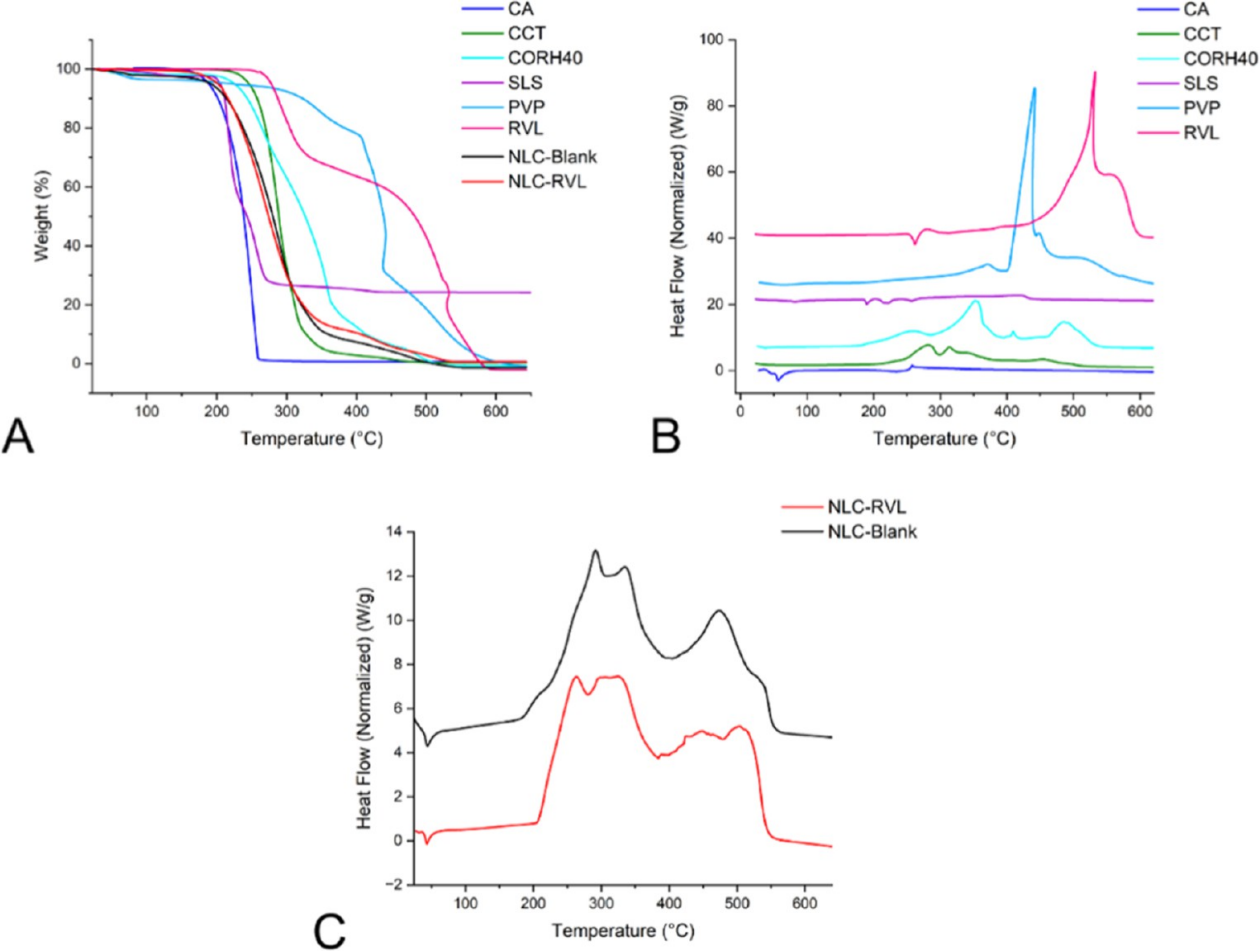
TG of raw materials, NLC-blank, and NLC-RVL (A), DSC of raw materials (B), and DSC of NLC-Blank and NLC-RVL (C).

The oil phase components—CA, CCT, and CORH40—constituted the major fraction of the formulation. CA exhibited two events: an endothermic melting peak at 56 °C with no mass loss, confirming its melting point,^39^ and decomposition starting at 150 °C, ending with an exothermic peak at 257 °C associated with oxidation due to the synthetic air atmosphere used. CCT began decomposition at ∼200 °C, completed at 350 °C, with two exothermic peaks (280 and 313 °C), and showed an exothermic peak at 453 °C with a small mass loss (4.5%), indicating oxidation of carbonaceous residues. CORH40 presented five events: a slightly pronounced endothermic melting at 37 °C (1.5% mass loss), decomposition starting at 200 °C with the main exothermic peak at 351 °C (>80% mass loss), and final oxidation up to 486 °C.

SLS displayed an endothermic peak at 81 °C (water loss) and melting at 190 °C.^40^ Between 200 and 300 °C, organic decomposition occurred, leaving 24% residue attributed to sodium sulfate (Na_2_SO_4_); the exothermic peak at 418 °C was related to elimination of carbonaceous material. PVP showed minor initial water loss, stability up to 300 °C, and pronounced degradation above 400 °C (80% mass loss), with a TG profile consistent with literature.^41^ RVL remained thermally stable up to ∼270 °C, with melting at 261 °C.^42,43^

For the formulations (Figure 11C), a small endothermic peak was observed at approximately 45 °C, consistent with stability studies that indicated instability at 40 °C due to proximity to the melting point. In both formulations, mass loss started at ∼100 °C, occurring almost entirely up to 400 °C, associated with degradation of organic components and marked by exothermic peaks.

When comparing NLC-Blank and NLC-RVL, a significant decrease in *T*_onset_ was observed (from 64 to 32 °C, respectively), along with higher mass loss for NLC-Blank (2.4%). Nevertheless, both exhibited a *T*_peak_ DSC of 45 °C, suggesting that the mass loss may be due to adsorbed water even after lyophilization. The *T*_peak_ DSC decreased from 292 °C (NLC-Blank) to 263 °C (NLC-RVL), indicating that RVL may accelerate thermal degradation. NLC-Blank presented a well-defined exothermic peak at 472 °C, whereas NLC-RVL displayed a different DSC profile in this range, producing more reactive residues that released energy at a higher temperature (*T*_peak_ DSC 502 °C).

#### 3.8.4 FTIR Spectroscopy

The components of the formulations were analyzed individually by FTIR, aiming at the structural characterization of each substance, with spectra recorded in the 4000–400 cm^–1^ range.

Cetostearyl alcohol (Figure S7A) exhibited characteristic absorption bands corresponding to the symmetric stretching of C–H bonds in methylene groups (−CH_2_) at 2916 cm^–1^, scissoring bending of −CH_2_ groups and asymmetric bending of CH_3_ groups at 1462 cm^–1^, rocking bending of the −CH_2_ group at 718 cm^–1^, and hydroxyl (−OH) stretching at 3284 cm^–1^.^44^

CCT (Figure S7B) showed characteristic aliphatic chain bands at 2922 cm^–1^ (CH_2_ stretching) and 2854 cm^–1^ (O–CH_2_ vibrations), a carbonyl (C=O) band at 1740 cm^–1^, (CH_2_)_*n*_ rocking at 721 cm^–1^ ^45^ and C–O–C stretching at 1153 cm^–1^.^46^

The surfactant CORH40 (Figure S7C) displayed OH stretching at 3502 cm^–1^, CH stretching at 2857 cm^–1^, C=O at 1732 cm^–1^, and ether C–O–C at 1098 cm^–1^.^47^ SLS (Figure S7D) presented −CH_2_ symmetric and asymmetric stretching bands at 2916 and 2848 cm^–1^, respectively, along with symmetric S=O stretching at 1214 and 1078 cm^–1^.^48^ Polyvinylpyrrolidone (Figure S7E) exhibited bands at 1661 cm^–1^ (C=O) and 1265 cm^–1^ (C–N).^49^

RVL (Figure S7F) displayed characteristic aromatic C=C stretching bands at 1605, 1583, and 1507 cm^–1^, hydroxyl (OH) at 1377 cm^–1^, and C=O at 1143 cm^–1^ (phenolic compounds). The band at 963 cm^–1^ corresponds to the CH group, indicating the trans configuration, while 827 cm^–1^ represents C–H vibration of the arene conjugated to an olefinic group, and 670 cm^–1^ corresponds to olefinic =C–H.^50^

The spectra of the formulations exhibited high similarity, as expected due to their comparable composition (Figure S7G). No distinct signals attributable to RVL were observed; the spectra were largely overlapping with bands at the same wavenumbers. This can be explained by the low concentration of RVL in the formulations, while the major components—lipids and surfactants—are present at much higher levels, dominating the spectral profile.

#### 3.8.5 Transmission Electron Microscopy

TEM analysis (Figure 12) revealed that NLC-RVL exhibited a spherical morphology, with particle sizes within the nanometric range, averaging approximately 50 nm in diameter—smaller than the size measured by DLS (90 nm).

**Figure 12:**
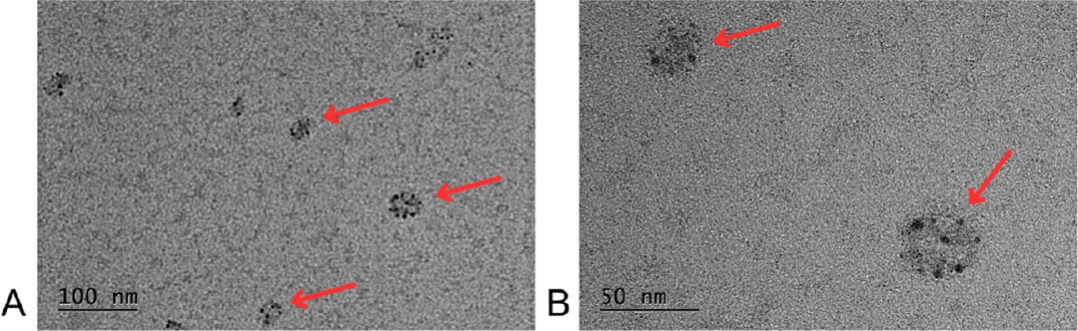
TEM of NLC-RVL at different magnifications. (A) 100 nm; (B) 50 nm. The arrows point to the lipid nanoparticles.

## 4 Conclusions

The lipid nanocarriers were successfully developed using the spontaneous emulsification method, a low-energy technique widely employed for producing nanoemulsions but still scarcely explored for lipid nanoparticles, which are predominantly obtained through high-energy methods. The application of the Box–Behnken statistical design enabled the optimization of both formulation and process parameters. Dispersion stability was enhanced by the addition of SLS, which imparted a negative surface charge to the particles. Conversely, replacing the dispersion polymer with P188 or increasing the PVP concentration did not yield significant benefits.

RVL was efficiently encapsulated (E.E. = 99%), exhibiting a sustained release profile. The final formulations, produced both in small batches and at a larger scale, in the presence or absence of RVL, showed particle sizes between 90 and 200 nm, PDI values ranging from 0.20 to 0.40, zeta potentials from −28 to −46 mV, and stability for up to 90 days at 8 °C. The formulations were thoroughly characterized, with RVL identified by DSC, quantified by spectrophotometry, and nanoscale size confirmed by TEM.

Given the broad spectrum of biological activities and health benefits associated with RVL, future research should explore the pharmaceutical and cosmetic potential of formulations through comprehensive in vitro, ex vivo, and in vivo evaluations. Additionally, unresolved aspects of the present study warrant further investigation, particularly the physical state of RVL within the lipid matrix, which should be elucidated by using X-ray diffraction analysis. Moreover, the investigation of extended sustained-release behavior represents an important area for future research, as the complete release of the encapsulated compound could not be conclusively demonstrated within the experimental time frame.

## Supplementary Material



## Notes

The calibration curve for UV–vis spectrophotometric determination of RVL, the preliminary stability study, the reproducibility test between the best formulations, Pareto charts revealing the significant factors in the composition and process, the quadratic regression model equations for formulation and process optimization, contour plots generated by RSM for different interactions between formulation composition variables and for different interactions between process variables, the composition of the final formulations at each stage of development, the two-way ANOVA for evaluating the effect of RVL addition and scaling, and the results of thermal analysis and FTIR spectra of raw materials and NLC formulations (PDF)

## References

[1] Musielak E., Feliczak-Guzik A., Nowak I. (2022). Synthesis and Potential Applications
of Lipid Nanoparticles in Medicine. Materials.

[2] Tenchov R., Bird R., Curtze A. E., Zhou Q. (2021). Lipid Nanoparticles–From
Liposomes to mRNA Vaccine Delivery, a Landscape of Research Diversity
and Advancement. ACS Nano.

[3] Montenegro L., Lai F., Offerta A., Sarpietro M. G., Micicchè L., Maccioni A. M., Valenti D., Fadda A. M. (2016). From Nanoemulsions
to Nanostructured Lipid Carriers: A Relevant Development in Dermal
Delivery of Drugs and Cosmetics. J. Drug Delivery
Sci. Technol..

[4] Ghasemiyeh P., Mohammadi-Samani S. (2018). Solid Lipid
Nanoparticles and Nanostructured Lipid
Carriers as Novel Drug Delivery Systems: Applications, Advantages
and Disadvantages. Res. Pharm. Sci..

[5] Souto E. B., Cano A., Martins-Gomes C., Coutinho T. E., Zielińska A., Silva A. M. (2022). Microemulsions and
Nanoemulsions in Skin Drug Delivery. Bioengineering.

[6] Akanda M., Mithu M. S. H., Douroumis D. (2023). Solid Lipid
Nanoparticles: An Effective
Lipid-Based Technology for Cancer Treatment. J. Drug Delivery Sci. Technol..

[7] Müller R. H., Shegokar R., Keck C. M. (2011). 20 Years
of Lipid Nanoparticles (SLN
& NLC): Present State of Development & Industrial Applications. Curr. Drug Discovery Technol..

[8] Aditya N. P., Macedo A. S., Doktorovova S., Souto E. B., Kim S., Chang P.-S., Ko S. (2014). Development
and Evaluation of Lipid
Nanocarriers for Quercetin Delivery: A Comparative Study of Solid
Lipid Nanoparticles (SLN), Nanostructured Lipid Carriers (NLC), and
Lipid Nanoemulsions (LNE). LWT—Food Sci.
Technol..

[9] Abid N., Khan A. M., Shujait S., Chaudhary K., Ikram M., Imran M., Haider J., Khan M., Khan Q., Maqbool M. (2022). Synthesis of Nanomaterials
Using
Various Top-down and Bottom-up Approaches, Influencing Factors, Advantages,
and Disadvantages: A Review. Adv. Colloid Interface
Sci..

[10] Gordillo-Galeano A., Mora-Huertas C. E. (2018). Solid Lipid
Nanoparticles and Nanostructured Lipid
Carriers: A Review Emphasizing on Particle Structure and Drug Release. Eur. J. Pharm. Biopharm..

[11] Kumar M., Bishnoi R. S., Shukla A. K., Jain C. P. (2019). Techniques
for Formulation
of Nanoemulsion Drug Delivery System: A Review. Prev. Nutr. Food Sci..

[12] Ganesan P., Narayanasamy D. (2017). Lipid Nanoparticles: Different Preparation
Techniques,
Characterization, Hurdles, and Strategies for the Production of Solid
Lipid Nanoparticles and Nanostructured Lipid Carriers for Oral Drug
Delivery. Sustainable Chem. Pharm..

[13] Gomaa E., Fathi H. A., Eissa N. G., Elsabahy M. (2022). Methods for
Preparation
of Nanostructured Lipid Carriers. Methods.

[14] PubChem . Resveratrol. https://pubchem.ncbi.nlm.nih.gov/compound/445154 (accessed Aug 14, 2025).

[15] Machado A.
C. H. R., Marinheiro L. J., Benson H. A. E., Grice J. E., Martins T. D. S., Lan A., Lopes P. S., Andreo-Filho N., Leite-Silva V. R. (2023). A Novel
Handrub Tablet Loaded with Pre- and Post-Biotic
Solid Lipid Nanoparticles Combining Virucidal Activity and Maintenance
of the Skin Barrier and Microbiome. Pharmaceutics.

[16] Losito D. W., Lopes P. S., Ueoka A. R., Fantini M. C. A., Oseliero
Filho P. L., Andréo-Filho N., Martins T. S. (2021). Biocomposites Based
on SBA-15 and Papain: Characterization, Enzymatic Activity and Cytotoxicity
Evaluation. Microporous Mesoporous Mater..

[17] Asadi J., Ferguson S., Raja H., Hacker C., Marius P., Ward R., Pliotas C., Naismith J., Lucocq J. (2017). Enhanced Imaging
of Lipid Rich Nanoparticles Embedded in Methylcellulose Films for
Transmission Electron Microscopy Using Mixtures of Heavy Metals. Micron.

[18] Taylor E. N., Kummer K. M., Dyondi D., Webster T. J., Banerjee R. (2014). Multi-Scale
Strategy to Eradicate Pseudomonas Aeruginosa on Surfaces Using Solid
Lipid Nanoparticles Loaded with Free Fatty Acids. Nanoscale.

[19] Nogueira N. C., De Sá L. L. F., De Carvalho A. L. M. (2022). Nanostructured Lipid Carriers as
a Novel Strategy for Topical Antifungal Therapy. AAPS PharmSciTech.

[20] Viegas C., Patrício A. B., Prata J. M., Nadhman A., Chintamaneni P. K., Fonte P. (2023). Solid Lipid Nanoparticles vs. Nanostructured Lipid Carriers: A Comparative
Review. Pharmaceutics.

[21] Assalem N., Abd-allah H., Ragaie M. H., Ahmed S. S., Elmowafy E. (2024). Therapeutic
Potential of Limonene-Based Syringic Acid Nanoemulsion: Enhanced Ex-Vivo
Cutaneous Deposition and Clinical Anti-Psoriatic Efficacy. Int. J. Pharm..

[22] Awasthi S., Hasan N., Nadeem M., Rizvi M. A., Alam K., Kesharwani P., Ahmad F. J. (2024). Optimized Formulation of Berberine
Hydrochloride Loaded Nanoemulgel for Management of Skin Cancer. Colloids Surf., A.

[23] Hasan N., Imran M., Nadeem M., Jain D., Haider K., Moshahid Alam Rizvi M., Sheikh A., Kesharwani P., Kumar Jain G., Jalees Ahmad F. (2023). Formulation and Development of Novel
Lipid-Based Combinatorial Advanced Nanoformulation for Effective Treatment
of Non-Melanoma Skin Cancer. Int. J. Pharm..

[24] Shirvani A., Goli S. A. H., Varshosaz J., Salvia-Trujillo L., Martín-Belloso O. (2022). Fabrication of Edible
Solid Lipid
Nanoparticle from Beeswax/Propolis Wax by Spontaneous Emulsification:
Optimization, Characterization and Stability. Food Chem..

[25] Loureiro
Contente D. M., Pereira R. R., Rodrigues A. M. C., Da Silva E. O., Ribeiro-Costa R. M., Carrera Silva-Júnior J. O. (2020). Nanoemulsions
of Acai Oil: Physicochemical Characterization for the Topical Delivery
of Antifungal Drugs. Chem. Eng. Technol..

[26] Ortiz A. C., Yañez O., Salas-Huenuleo E., Morales J. O. (2021). Development of a
Nanostructured Lipid Carrier (NLC) by a Low-Energy Method, Comparison
of Release Kinetics and Molecular Dynamics Simulation. Pharmaceutics.

[27] Mishra V., Bansal K. K., Verma A., Yadav N., Thakur S., Sudhakar K., Rosenholm J. M. (2018). Solid Lipid
Nanoparticles: Emerging
Colloidal Nano Drug Delivery Systems. Pharmaceutics.

[28] Rachmawati H., Novel M., Ayu S., Berlian G., Tandrasasmita O., Tjandrawinata R., Anggadiredja K. (2017). The In Vitro–In Vivo Safety
Confirmation of PEG-40 Hydrogenated Castor Oil as a Surfactant for
Oral Nanoemulsion Formulation. Sci. Pharm..

[29] Jintapattanakit A. (2018). Preparation
of Nanoemulsions by Phase Inversion Temperature (PIT). Pharm. Sci. Asia.

[30] Abdelhameed A. H., Abdelhafez W. A., Saleh K. H. I., Mohamed M. S. (2022). Formulation,
Optimization,
and in-Vivo Evaluation of Nanostructured Lipid Carriers Loaded with
Fexofenadine HCL for Oral Delivery. J. Drug
Delivery Sci. Technol..

[31] Dhiman N., Awasthi R., Sharma B., Kharkwal H., Kulkarni G. T. (2021). Lipid Nanoparticles
as Carriers for Bioactive Delivery. Front. Chem..

[32] Basso J., Mendes M., Cova T., Sousa J., Pais A., Fortuna A., Vitorino R., Vitorino C. (2022). A Stepwise
Framework
for the Systematic Development of Lipid Nanoparticles. Biomolecules.

[33] Joshy K. S., Snigdha S., Anne G., Nandakumar K., Pothen L. A., Sabu T. (2018). Poly (Vinyl Pyrrolidone)-Lipid
Based
Hybrid Nanoparticles for Anti Viral Drug Delivery. Chem. Phys. Lipids.

[34] Chen W. N., Shaikh M. F., Bhuvanendran S., Date A., Ansari M. T., Radhakrishnan A. K., Othman I. (2022). Poloxamer 188 (P188), A Potential
Polymeric Protective Agent for CentralNervous System Disorders: A
Systematic Review. Curr. Neuropharmacol..

[35] Chantaburanan T., Teeranachaideekul V., Jintapattanakit A., Chantasart D., Junyaprasert V. B. (2023). Enhanced
Stability and Skin Permeation of Ibuprofen-Loaded
Solid Lipid Nanoparticles Based Binary Solid Lipid Matrix: Effect
of Surfactant and Lipid Compositions. Int. J.
Pharm.: X.

[36] Gómez-Lázaro L., Martín-Sabroso C., Aparicio-Blanco J., Torres-Suárez A. I. (2024). Assessment of In
Vitro Release Testing
Methods for Colloidal Drug Carriers: The Lack of Standardized Protocols. Pharmaceutics.

[37] Robinson K., Mock C., Liang D. (2015). Pre-Formulation
Studies of Resveratrol. Drug Dev. Ind. Pharm..

[38] Radeva L., Yoncheva K. (2025). Resveratrol—A
Promising Therapeutic Agent with
Problematic Properties. Pharmaceutics.

[39] Seif M., Impelido M. L., Apps M. G., Wheate N. J. (2014). Topical Cream-Based
Dosage Forms of the Macrocyclic Drug Delivery Vehicle Cucurbit[6]­Uril. PLoS One.

[40] Al-Mohizea A. M., Al-Jenoobi F. I., Alam M. A. (2015). Rhodamine-123: A
p-Glycoprotein Marker
Complex with Sodium Lauryl Sulfate. Pak. J.
Pharm. Sci..

[41] Salles T.
H. C., Lombello C. B., d’Ávila M. A. (2015). Electrospinning
of Gelatin/Poly (Vinyl Pyrrolidone) Blends from Water/Acetic Acid
Solutions. Mater. Res..

[42] Ainurofiq A., Hidayat Y., Lestari E. Y. P., Kumalasari M. M. W., Choiri S. (2022). Resveratrol Nanocrystal Incorporated into Mesoporous
Material: Rational Design and Screening through Quality-by-Design
Approach. Nanomaterials.

[43] Todan L., Voicescu M., Culita D. C., Pandele-Cuşu J., Albu C., Kuncser A. C. (2021). Ecological Formulation for Improving
Resveratrol Stability and Release in Aqueous Environment. Chem. Pap..

[44] Narvaez L. E. M., Carrillo M. P., Cardona-Jaramillo J.
E. C., Vallejo B. M., Ferreira L. M. D. M. C., Silva-Júnior J.
O. C., Ribeiro-Costa R. M. (2023). Novel Organogels
from Mauritia Flexuosa L.f and Caryodendron Orinocense Karst.: A Topical
Alternative. Pharmaceutics.

[45] Guilherme V. A., Ribeiro L. N. M., Alcântara A. C.
S., Castro S. R., Rodrigues Da Silva G. H., Da Silva C. G., Breitkreitz M. C., Clemente-Napimoga J., Macedo C. G., Abdalla H. B., Bonfante R., Cereda C. M. S., De Paula E. (2019). Improved Efficacy of Naproxen-Loaded
NLC for Temporomandibular Joint Administration. Sci. Rep..

[46] Ye K., Zhao D., Shi X., Lu X. (2016). Use of Caprylic/Capric
Triglyceride in the Encapsulation of Dementholized Peppermint Fragrance
Leading to Smaller and Better Distributed Nanocapsules. RSC Adv..

[47] Kraisit P., Hirun N., Limpamanoch P., Sawaengsuk Y., Janchoochai N., Manasaksirikul O., Limmatvapirat S. (2024). Effect of
Cremophor RH40, Hydroxypropyl Methylcellulose, and Mixing Speed on
Physicochemical Properties of Films Containing Nanostructured Lipid
Carriers Loaded with Furosemide Using the Box–Behnken Design. Polymers.

[48] Jessima S. J. H. M., Berisha A., Srikandan S. S. S., Subhashini S. (2020). Preparation,
characterization, and Evaluation of Corrosion Inhibition Efficiency
of Sodium Lauryl Sulfate Modified Chitosan for Mild Steel in the Acid
Pickling Process. J. Mol. Liq..

[49] Hung Y.-C., Hsieh S.-C., Hou S.-R., Kung J.-Y., Tang C.-M., Chang C.-J. (2021). In Vivo Evaluation of PVP-Gelatin-Chitosan
Composite
Blended with Egg-Yolk Oil for Radiodermatitis. Appl. Sci..

[50] Jermy B. R., Al-Jindan R. Y., Ravinayagam V., El-Badry A. A. (2022). Anti-Blastocystosis
Activity of Antioxidant Coated ZIF-8 Combined with Mesoporous Silicas
MCM-41 and KIT-6. Sci. Rep..

