# Supplementary material for: Spontaneous Emulsification as a Low-Energy Strategy for Designing and Optimizing Resveratrol-Loaded Nanostructured Lipid Carriers
Source: ACS Omega. 2026 Feb 11;11(7):11833–50. doi: 10.1021/acsomega.5c10613 (PMC12947224; doi:10.1021/acsomega.5c10613)
Supplement: Supplementary file 1 [file ao5c10613_si_001.pdf]

Spontaneous emulsification as a low-energy strategy for designing and optimizing Resveratrol-loaded Nanostructured Lipid Carriers

Nicoly T. R. Britto [a]; Lilian R. S. Montanheri [a]; Juliane N. B. D. Pelin [a]; Tereza S. Martins [b]; Patrícia S. Lopes [a,*]; Vânia R. Leite-Silva [a,c]; Newton Andreo-Filho [a]

[a] Department of Pharmaceutical Sciences, Federal University of Sao Paulo, Diadema 09913-030, SP, Brazil;
[b] Department of Chemistry, Federal University of Sao Paulo, Diadema 09913-030, SP, Brazil
[c] Therapeutics Research Centre, The University of Queensland Diamantina Institute, Translational Research Institute, Brisbane, QL 4102, Australia
*Corresponding author: patricia.lopes@unifesp.br

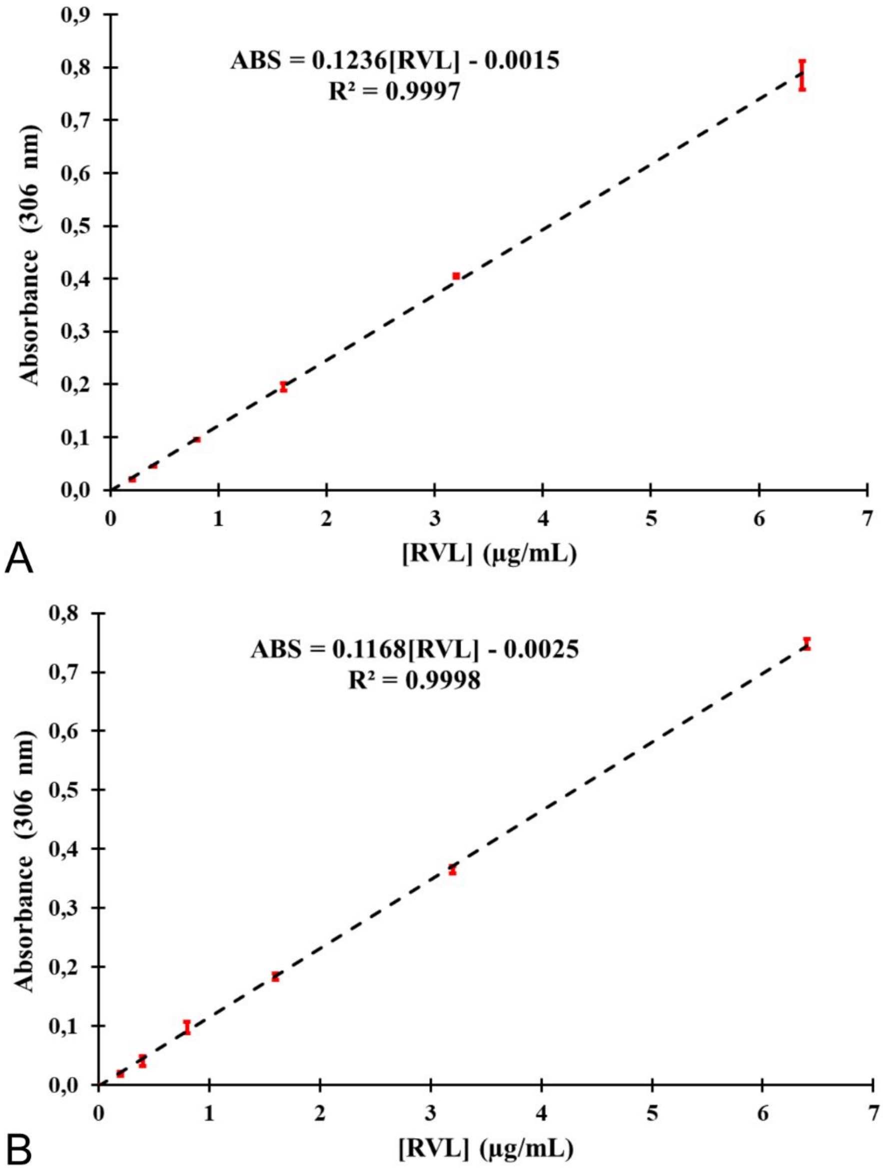

Figure S1. Calibration curve for UV-vis spectrophotometric determination of resveratrol in A- etanol, B- PBS with 0.5% w/v Tween 80. ABS: absorbance, [RVL]: resveratrol concentration

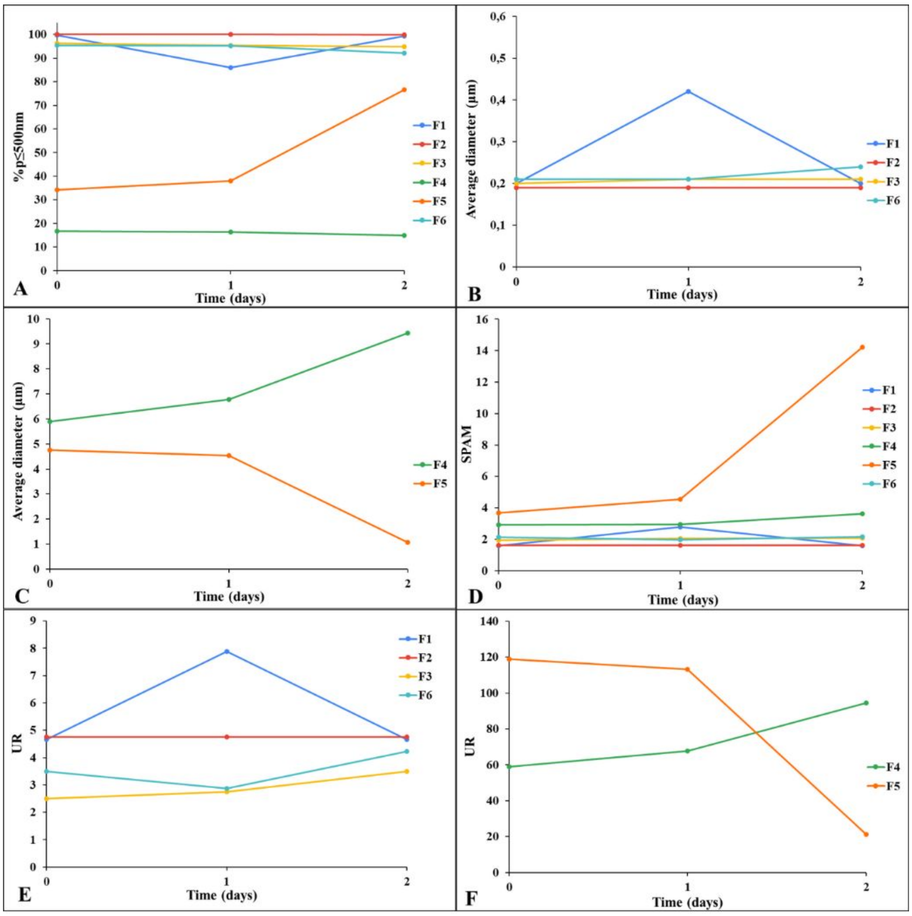

Figure S2. Preliminary stability study regarding the PSD of formulations (A) %p≤500nm (B) Average diameter (µm) (C) Average diameter (µm) F4 and F5 (D) SPAM (E) UR (E) UR F4 and F5

Table S1. Reproducibility test between the best formulations

Evaluated parameters	F0		FR		Variation (%)
%p≤500nm ± SD	F1	99.76 ± 0.04	F1R	94.83 ± 0.18	4.9
	F2	100 ± 0.0	F2R	99.48 ± 0.10	0.5
	F3	96.25 ± 0.01	F3R	74.66 ± 0.19	22.4
	F6	95.44 ± 0.11	F6R	94.29 ± 0.25	1.2
Average Diameter (µm) ± SD	F1	0.20 ± 0.00	F1R	0.21 ± 0.00	5
	F2	0.19 ± 0.00	F2R	0.20 ± 0.00	5.3
	F3	0.20 ± 0.00	F3R	0.80 ± 0.01	301.7
	F6	0.21 ± 0.00	F6R	0.22 ± 0.00	4.8
SPAM ± SD	F1	1.58 ± 0.00	F1R	2.08 ± 0.03	31.6
	F2	1.61 ± 0.00	F2R	1.52 ± 0.03	5.9
	F3	1.94 ± 0.00	F3R	9.76 ± 0.10	404
	F6	2.13 ± 0.00	F6R	2.14 ± 0.03	0.6
UR ± SD	F1	4.67 ± 0.58	F1R	3.73 ± 0.40	20
	F2	4.75 ± 0.00	F2R	5.00 ± 0.00	5.3
	F3	2.50 ± 0.00	F3R	16.07 ± 0.12	542.7
	F6	3.50 ± 0.00	F6R	3.67 ± 0.00	4.8

%p≤500nm ± SD: percentage of particles with diameter less than or equal to 500 nm ± standard deviation; SPAM ± SD: dispersity ± standard deviation; UR ± SD: Uniformity Ratio ± standard deviation; F0: Initial Formulation; FR: Repeated Formulation.

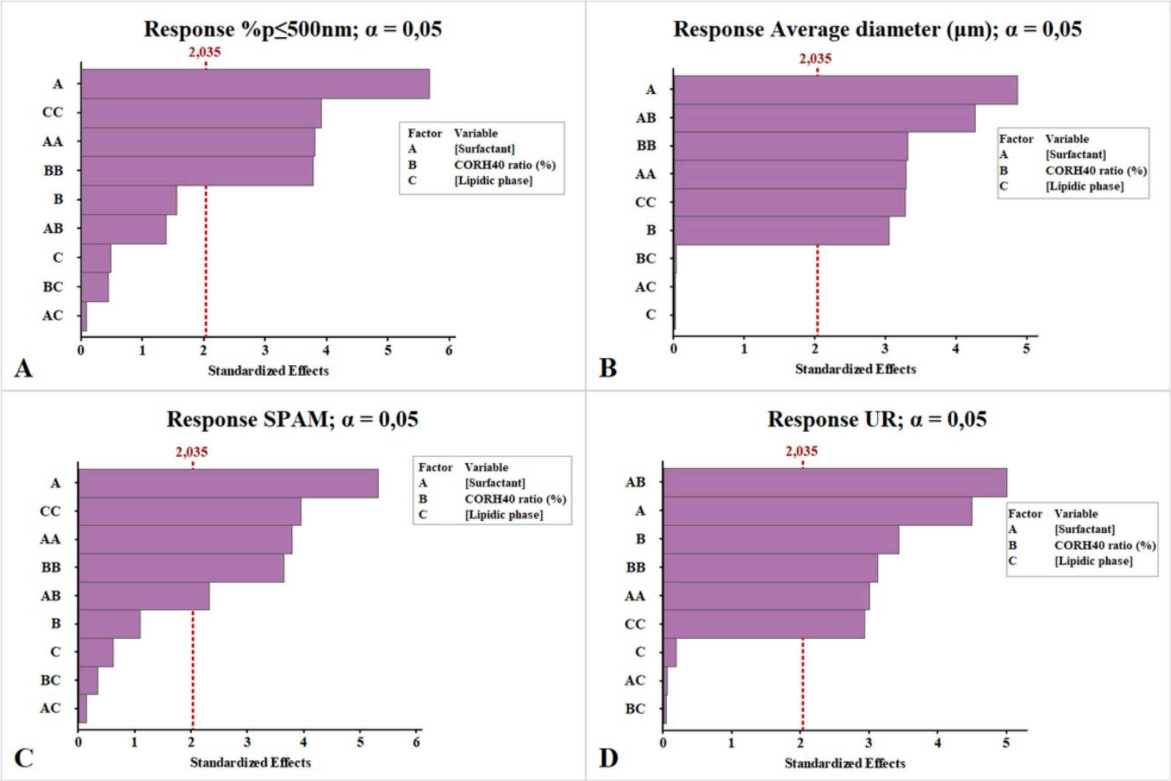

Figure S3. Pareto charts revealing the significant factors in the composition of the formulations for each PSD response (A) %p≤500nm (B) Average diameter (µm) (C) SPAM (D) UR

Table S2. Quadratic regression model equations – DoE for formulation optimization

Response	Quadratic Regression Equations
%p≤500nm =	$4.2 + 41.76\ X_1 + 0.886\ X_2 - 26.24\ X_3$<br>$- 3.306\ X_1^2 - 0.00525\ X_2^2 + 3.399\ X_3^2$<br>$- 0.0459\ X_1{*}X_2 + 0.070\ X_1{*}X_3 - 0.0146\ X_2{*}X_3$
Average diameter (μm) =	$7.22 - 2.671\ X_1 - 0.0853\ X_2 + 1.475\ X_3$<br>$+ 0.1844\ X_1^2 + 0.000297\ X_2^2 - 0.1842\ X_3^2$<br>$+ 0.00921\ X_1{*}X_2 - 0.0010\ X_1{*}X_3 + 0.00005\ X_2{*}X_3$
SPAM=	$7.65 - 3.90\ X_1 - 0.0125\ X_2 + 2.997\ X_3$<br>$+ 0.3686\ X_1^2 + 0.000567\ X_2^2 - 0.3846\ X_3^2$<br>$- 0.00869\ X_1{*}X_2 - 0.0128\ X_1{*}X_3 + 0.00126\ X_2{*}X_3$
UR=	$179.9 - 63.7\ X_1 - 2.294\ X_2 + 33.6\ X_3$<br>$+ 4.20\ X_1^2 + 0.00697\ X_2^2 - 4.10\ X_3^2$<br>$+ 0.2690\ X_1{*}X_2 - 0.07\ X_1{*}X_3 - 0.0023\ X_2{*}X_3$

X_1= [Surfactant]; X_2: CORH40 ratio (%); X_3= [Lipidic phase]

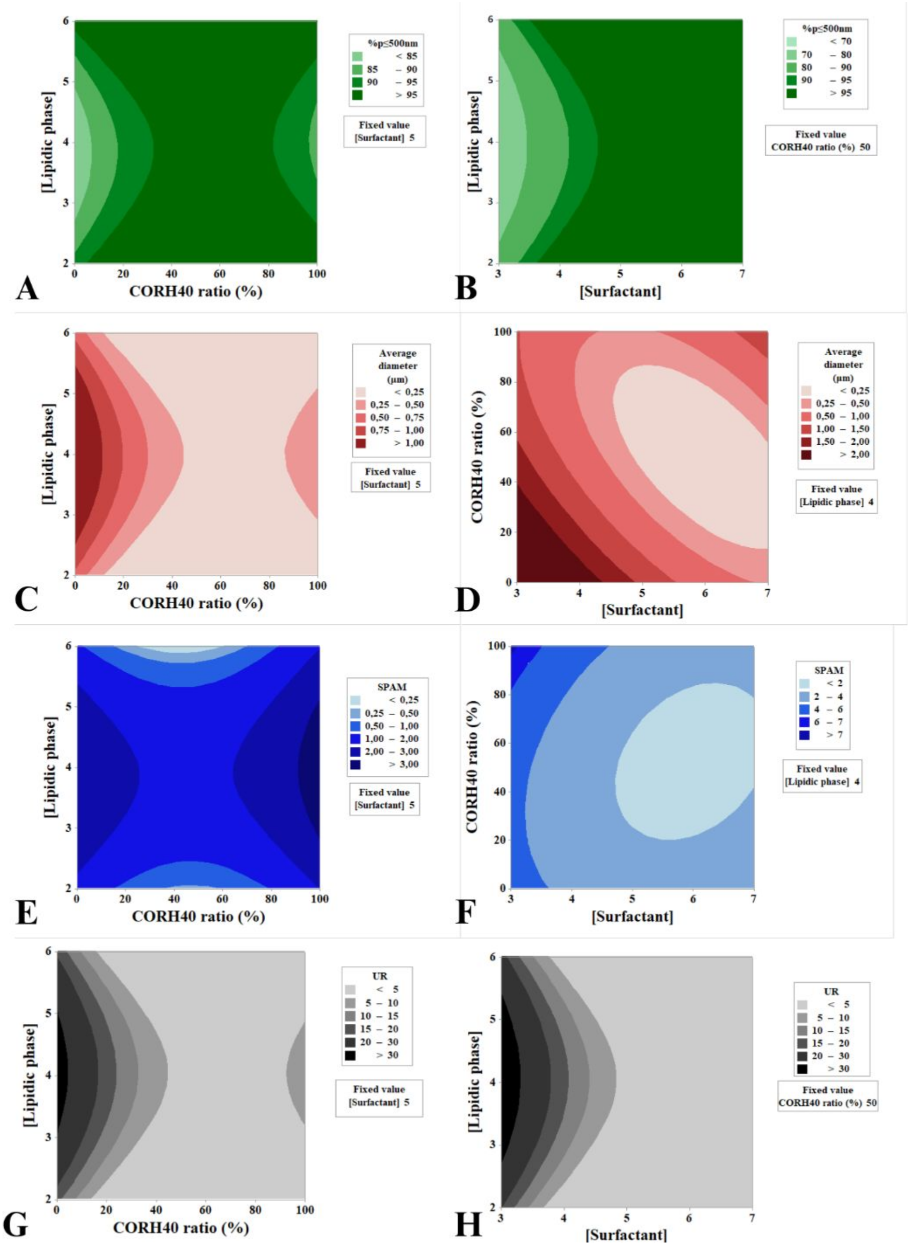

Figure S4. Contour plots generated by RSM for different interactions between formulation composition variables to optimize PSD values (A) and (B) %p≤ 500nm, (C) and (D) average diameter (µm), (E) and (F) SPAM, (G) and (H) UR

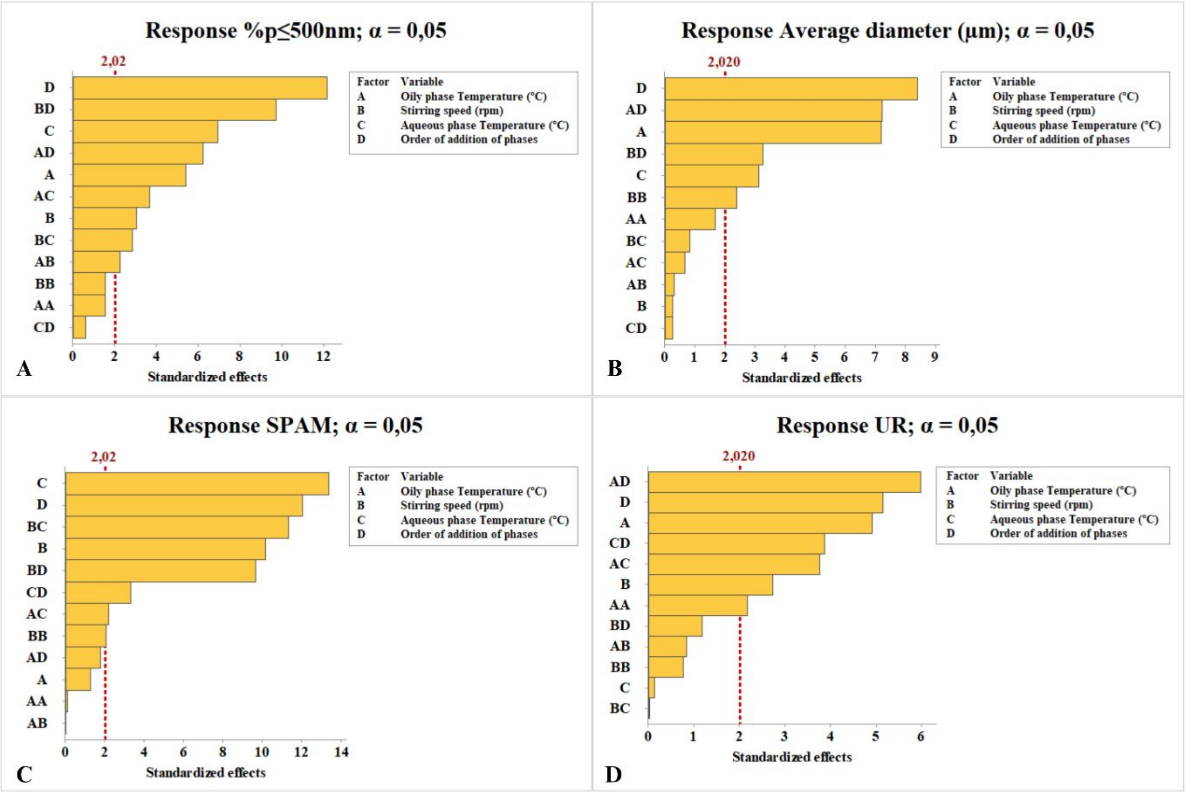

Figure S5. Pareto charts showing the significant process factors for each response related to particle size distribution (A) %p≤500nm (B) Average diameter (μm) (C) SPAM (D) UR

Table S3. Quadratic Regression Model Equations – DoE for Process Optimization

Order of addition of phases	Response	Quadratic Regression Equations
Aqueous over oily	%p≤500nm =	$-3682 + 82.9 X_1 - 0.967 X_2 + 29.85 X_3 - 0.263 X_1^2 + 0.000331 X_2^2 - 0.00809 X_1{*}X_2 - 0.524 X_1{*}X_3 + 0.01200 X_2{*}X_3$
Oily over aqueous		$-3445 + 77.0 X_1 - 0.696 X_2 + 29.21 X_3 - 0.263 X_1^2 + 0.000331 X_2^2 - 0.00809 X_1{*}X_2 - 0.524 X_1{*}X_3 + 0.01200 X_2{*}X_3$
Aqueous over oily	Average diameter (µm) =	$284 - 8.43 X_1 + 0.1548 X_2 - 1.07 X_3 + 0.0529 X_1^2 - 0.000095 X_2^2 + 0.000210 X_1{*}X_2 + 0.0182 X_1{*}X_3 -0.000659 X_2{*}X_3$
Oily over aqueous		$214 - 7.15 X_1 + 0.1378 X_2 - 1.02 X_3 + 0.0529 X_1^2 - 0.000095 X_2^2 + 0.000210 X_1{*}X_2 + 0.0182 X_1{*}X_3 - 0.000659 X_2{*}X_3$
Aqueous over oily	SPAM=	$-1265 + 9.80 X_1 + 1.527 X_2 + 15.23 X_3 + 0.0077 X_1^2 + 0.000175 X_2^2 + 0.00002 X_1{*}X_2 - 0.1253 X_1{*}X_3 - 0.01892 X_2{*}X_3$
Oily over aqueous		$-1296 + 9.13 X_1 + 1.419 X_2 + 16.60 X_3 + 0.0077 X_1^2 + 0.000175 X_2^2 + 0.00002 X_1{*}X_2 - 0.1253 X_1{*}X_3 - 0.01892 X_2{*}X_3$
Aqueous over oily	UR=	$15795 - 319.4 X_1 - 1.02 X_2 - 120.6 X_3 + 1.243 X_1^2 + 0.000541 X_2^2 + 0.0101 X_1{*}X_2 + 1.813 X_1{*}X_3 - 0.0003 X_2{*}X_3$
Oily over aqueous		$13566 - 300.3 X_1 - 1.13 X_2 - 107.2 X_3 + 1.243 X_1^2 + 0.000541 X_2^2 + 0.0101 X_1{*}X_2 + 1.813 X_1{*}X_3 - 0.0003 X_2{*}X_3$

X_1= Oily phase Temperature (ºC); X_2: Stirring speed (rpm); X_3= Aqueous phase Temperature (ºC)

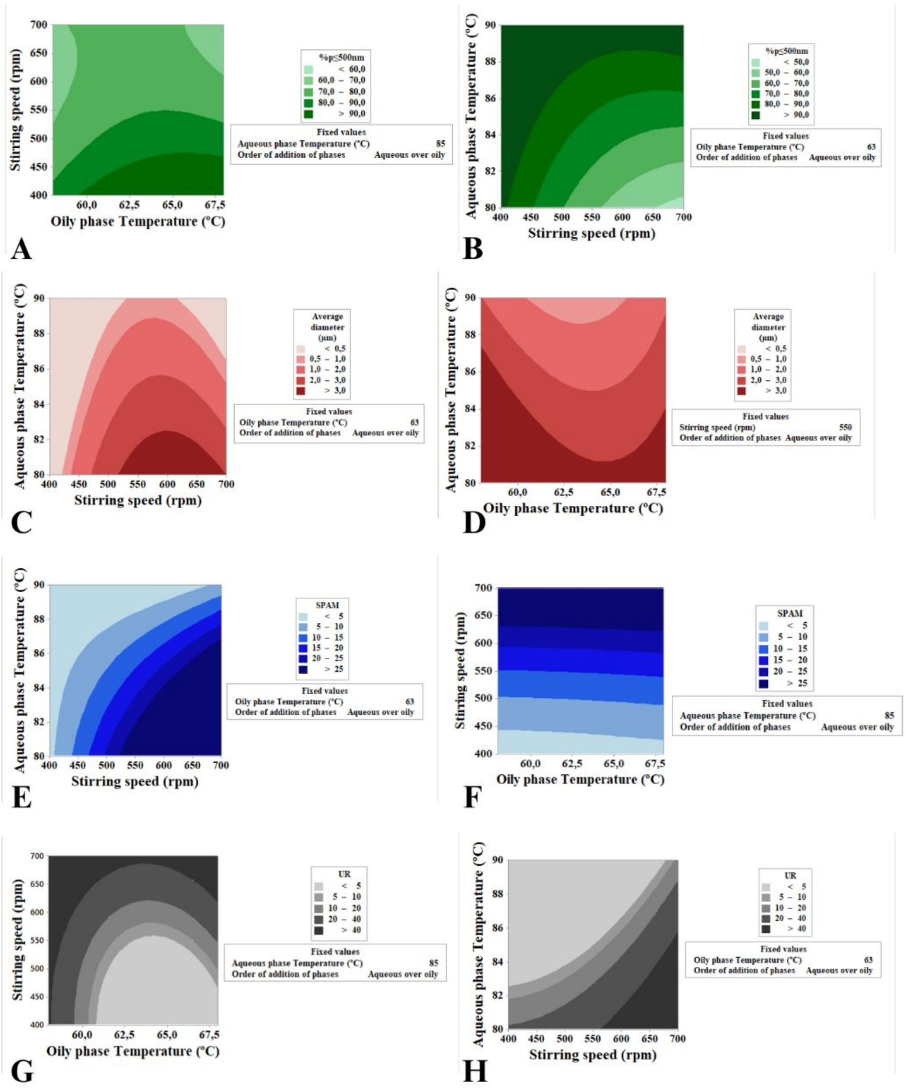

Figure S6. Contour plots generated by RSM for different interactions between process variables to optimize PSD values. (A) and (B) %p≤ 500nm. (C) and (D) average diameter (µm). (E) and (F) SPAM. (G) and (H) UR

Table S4. Composition of the final formulations at each stage of development

Development Step ➔		1- Selection of solid lipid and surfactant		2 and 3- Formulation optimization	4- Surface ionic charge addition	5- Resveratrol Incorporation
Selected/final formulation ➔		F2	F6	OFE	F1/NLC-Blank	NLC-RVL
Components		Concentration (% w/w)				
Lipidic phase	CA	2		2,85	2,85	2,85
	GS		2			
	CCT	2	2	2,85	2,85	2,85
	CORH40		5	5,9	5,9	5,9
	C20	5				
	RVL solution 5%					0,5
Aqueous phase	SLS				0,05	0,05
	PVP 2%	50	50	50	50	50
	Water q.s	100	100	100	100	100

CA: Cetearyl Alcohol; GS: Glyceryl Stearate; CCT: Caprylic/Capric Triglyceride; CORH40: PEG-40 Hydrogenated Castor Oil; C20: Ceteareth-20; RVL: Resveratrol; SLS: Sodium Lauryl Sulfate; PVP: Polyvinylpyrrolidone K30

Table S5. Two-way ANOVA for evaluating the effect of resveratrol addition and scaling on particle size. PDI. and ZP

Parameter	ANOVA two-way				Tukey			
	Factor	F value	P value	model R^2 (%)	Factor		Mean	Groups
**Size (nm)**	Scale	24.46	0.0001	64.38	Scale	Scaled-up	212.16	A
						Standard	91.94	B
	RVL	2.47	0.1354		RVL	No	171.15	A
						Yes	132.94	A
	Scale*RVL	1.99	0.1777		Scale-RVL	Scaled-up-No	248.4	A
						Scaled-up-Yes	175.92	A - B
						Standard-No	93.91	B
						Standard-Yes	89.97	B
**PDI**	Scale	10.43	0.0052	48.98	Scale	Scaled-up	0.4159	A
						Standard	0.2773	B
	RVL	2.04	0.1729		RVL	No	0.3772	A
						Yes	0.316	A
	Scale*RVL	2.89	0.1085		Scale-RVL	Scaled-up-No	0.4829	A
						Scaled-up-Yes	0.3488	A - B
						Standard-Yes	0.2832	B
						Standard-No	0.2714	B
**ZP (mV)**	Scale	28.67	0.0006	83.14	Scale	Standard	-32.9	A
						Scaled-up	-44.4	B
	RVL	0.9	0.3696		RVL	No	-37.6	A
						Yes	-39.7	A
	Scale*RVL	9.88	0.0137		Scale-RVL	Standard-No	-28.5	A
						Standard-Yes	-37.3	A - B
						Scaled-up-Yes	-42.1	B
						Scaled-up-No	-46.8	B

Table S6. Results of thermal analysis (TGA/DTG and DSC) of raw materials and NLC formulations

Steps	Parameters	ACE	TCC	CORH40	LSS	PVP	RVL	NLC-blank	NLC-RVL
	Temperature Range (°C)	25 - 150	25 - 350	25 - 76	25 - 200	25 - 100	25 - 350	25 - 150	25- 150
	Δw (%)	0.2	96.7	1.5	2.8	3.7	33	2.4	0.8
1	T_{onset} (°C)	81	267	43	190	40	271	64	32
	T_{peak} DTG (°C)	81	288	36	-	56	293	68	46
	T_{peak} DSC (°C)	56 (□)	280 (□) 313 (□)	37 (□)	81 (□) 190 (□)	-	261 (□)	45 (□)	45 (□)
	Temperature Range (°C)	150 - 650	350 - 650	76 - 290	200 - 236	100 - 400	350 - 650	150 - 400	150 - 400
	Δw (%)	101	4.5	31.3	43	18.5	70	90.2	88.9
2	T_{onset} (°C)	226	357	234	212	296	499	236	221
	T_{peak} DTG (°C)	251	-	267	218	343	531	292	269
	T_{peak} DSC (°C)	235 (□) 257 (□)	453 (□)	259 (□)	221 (□)	369 (□)	532 (□)	292 (□)	263 (□)
	Temperature Range (°C)			290 - 390	236 - 650	400 - 650		400 - 650	400 - 650
	Δw (%)			53.1	26.7	81		8.7	10
3	T_{onset} (°C)			339	254	444		451	423
	T_{peak} DTG (°C)			358	258	441		475	518
	T_{peak} DSC (°C)			351 (□)	256 (□) 418 (□)	441 (□)		472 (□)	502 (□)
	Temperature Range (°C)			390 - 459					
	Δw (%)			8.5					
4	T_{onset} (°C)			404					
	T_{peak} DTG (°C)			409					
	T_{peak} DSC (°C)			409 (□)					
	Temperature Range (°C)			459 - 650					
	Δw (%)			6.7					
5	T_{onset} (°C)			478					
	T_{peak} DTG (°C)			489					
	T_{peak} DSC (°C)			486 (□)					
	Resíduo em 650 °C (Δw %)	0.63	0.35	0	24	0	0	0	0.6

T: temperature; Δw: weight loss; □: exothermic peak; □: endothermic peak; ACE- Cetearyl Alcohol; TCC- Caprylic/Capric Triglyceride; CORH40- PEG-40 Hydrogenated Castor Oil; LSS- Sodium Lauryl Sulfate; PVP- Polyvinylpyrrolidone K30; RVL- Resveratrol

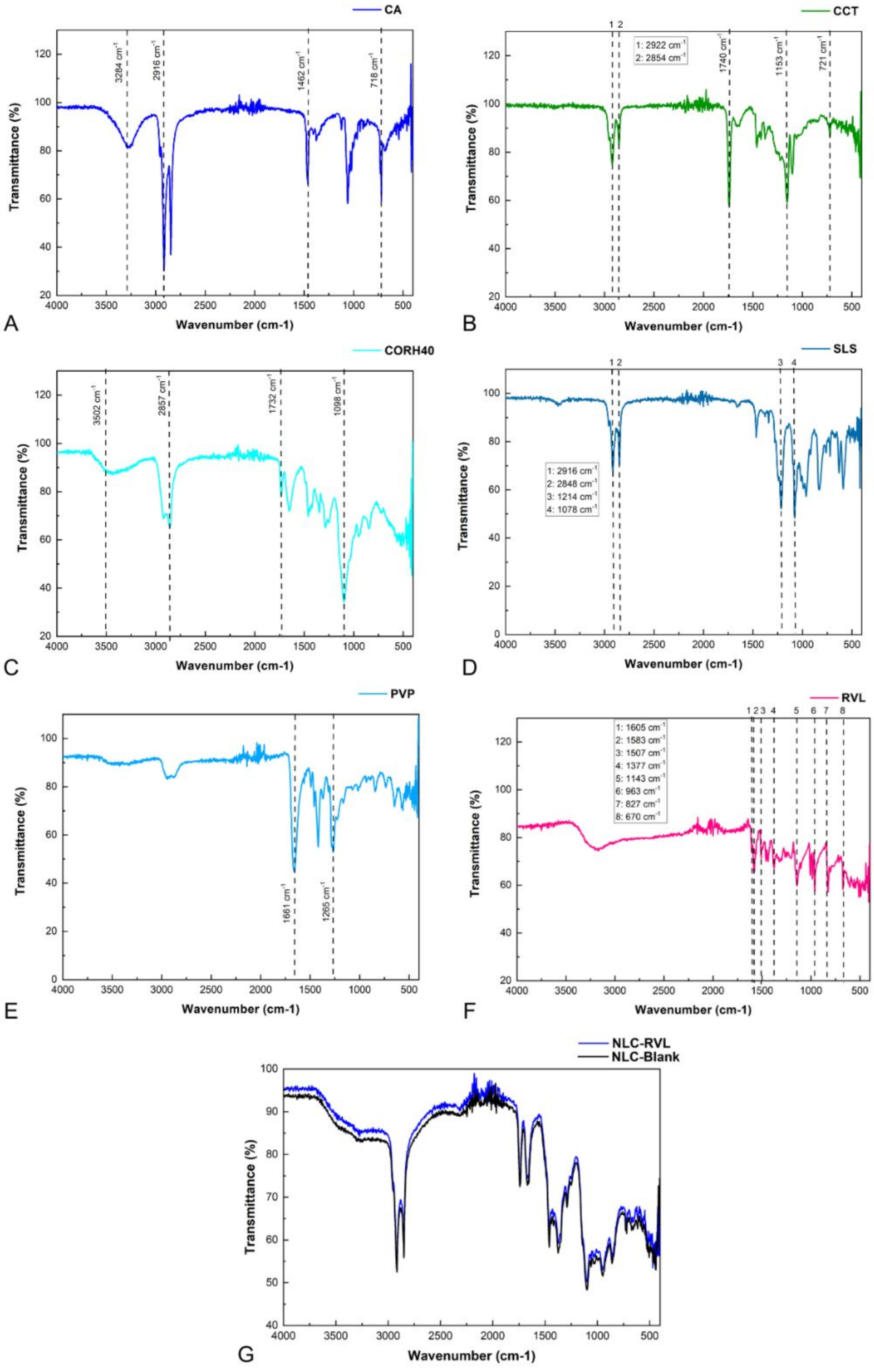

Figure S7. FTIR spectra of raw materials and NLC formulations. (A) Cetearyl Alcohol (CA); (B) Caprylic/Capric Triglyceride (CCT); (C) PEG-40 Hydrogenated Castor Oil (CORH40); (D) Sodium Lauryl Sulfate (SLS); (E) Polyvinylpyrrolidone K30 (PVP); (F) Resveratrol (RVL); (G) NLC-Blank (black line) NLC-RVL (blue line).
